# Supplementary material for: YAP-driven malignant reprogramming of oral epithelial stem cells at single cell resolution
Source: Nat Commun. 2025 Jan 8;16:498. doi: 10.1038/s41467-024-55660-6 (PMC11711616; doi:10.1038/s41467-024-55660-6)
Supplement: Supplementary file 1 — Supplementary Information [file 41467_2024_55660_MOESM1_ESM.pdf]

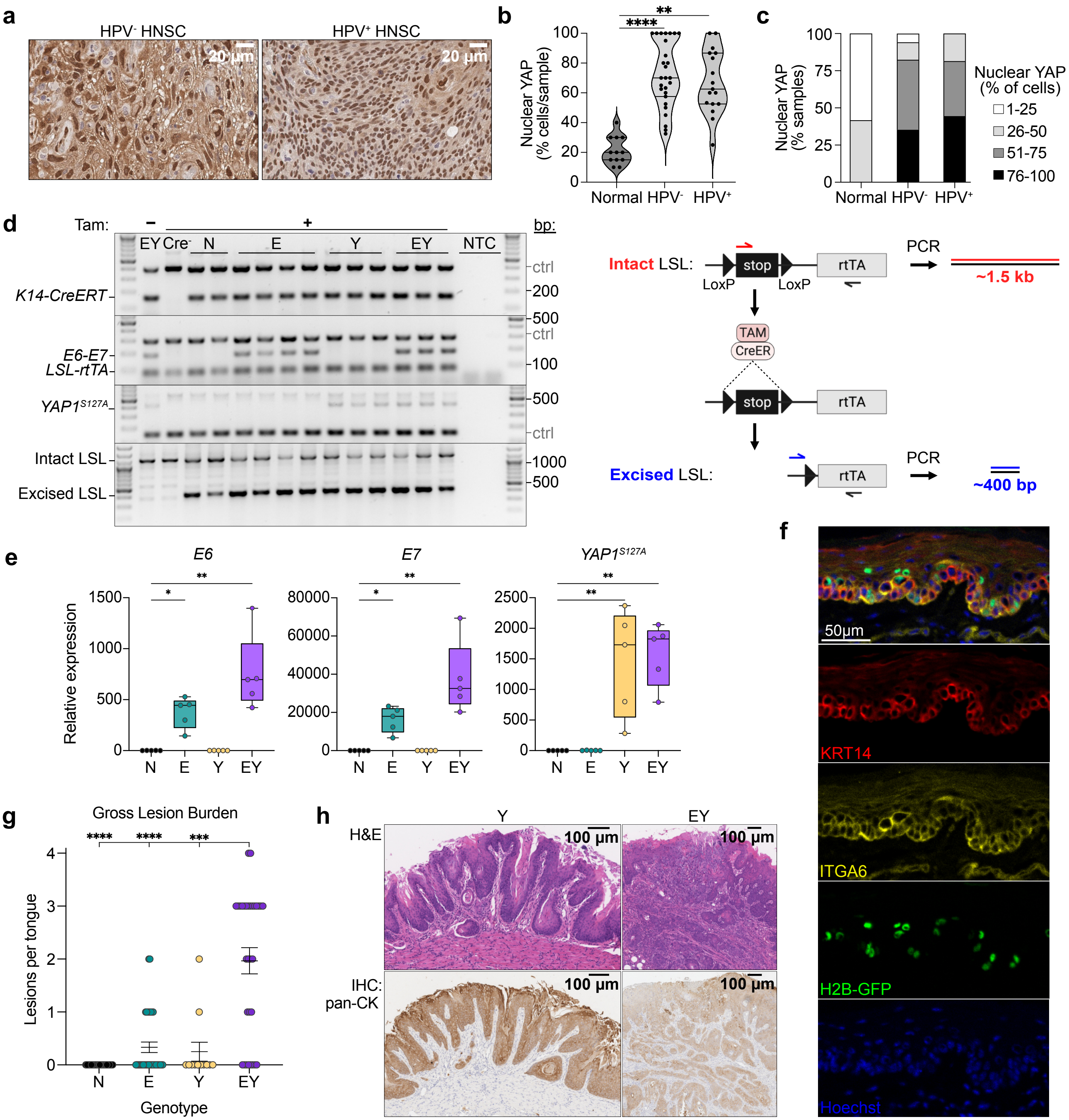
a
HPV- HNSC
HPV+ HNSC
20 µm
b
Nuclear YAP (% cells/sample)
Normal HPV- HPV+
c
Nuclear YAP (% samples)
Nuclear YAP (% of cells)
1-25
26-50
51-75
76-100
d
Tam:
EY Cre- N E Y EY NTC
bp:
K14-CreERT
E6-E7
LSL-rtTA
YAP1S127A
Intact LSL
Excised LSL
ctrl
Intact LSL:
stop
rtTA
LoxP
PCR
~1.5 kb
TAM
CreER
Excised LSL:
~400 bp
e
E6
E7
YAP1S127A
Relative expression
N E Y EY
f
50µm
KRT14
ITGA6
H2B-GFP
Hoechst
g
Gross Lesion Burden
Lesions per tongue
Genotype
h
H&E
IHC: pan-CK
100 µm

Supplementary Figure 1. Spatiotemporally controlled YAP and E6-E7 activation in OEPCs
(a) Representative images of nuclear YAP protein by IHC in HPV negative (left) and HPV positive (right) human HNSC; scale bars both 20μm. (b) Percent of cells with nuclear YAP in normal human oral or oropharyngeal epithelium (n=12) and HNSC stratified by HPV status (n=27 HPV^-, 17 HPV^+). Two-tailed Mann-Whitney test: **$p<0.01$, ****$p<0.0001$. (c) Percent of samples with positive nuclear YAP in normal oral or oropharyngeal epithelium (n=12) and HNSC (n=27 HPV^-, 17 HPV^+). (d) Left: Genomic DNA extracted from tongue epithelia 10 days after transgene induction with tamoxifen and doxycycline treatment. Top three gel images show presence of transgene and internal control (ctrl) PCR products. Bottom gel image confirms Lox_p-Stop-Lox_p (LSL) recombination for *LSL-rtTA*. Lane 1 mouse tongue epithelial gDNA from a tamoxifen and doxycycline untreated mouse bearing all transgenes. NTC, no template control. Right: Schematic of oligonucleotide primer design to assay LSL excision. (e) qRT-PCR quantification of HPV^{E6}, HPV^{E7}, and YAP^{S127A} expression in tongue epithelia 10 days after transgene induction. Boxplots show median, interquartile range (IQR), and range. (f) Immunofluorescence image of tongue epithelia from a $Krt14\text{-}CreER^+LSL\text{-}rtTA^+tetON\_H2B\text{-}GFP^+$ mouse 6 days after transgene induction showing basal localization of KRT14 and ITGA6, and nuclear expression of H2B-GFP. Nuclei are counterstained with Hoescht. Scale bars all 50μm. Images representative of 5 biological replicates. (g) Total number of lesions per tongue 20 days after transgene induction. Means with SEM are shown. (h) Hematoxylin and eosin (top) and pan-cytokeratin (pan-CK) IHC (bottom) stained tongue epithelia demonstrating infiltrative carcinoma in Y (left panels) and EY (right panels) mice; scale bars all 100μm. For panels h-k: N=14, E=11, Y=11, EY=16 mice per group. Source Data is available for panels b, c, e, and g.
Related to Fig. 1.

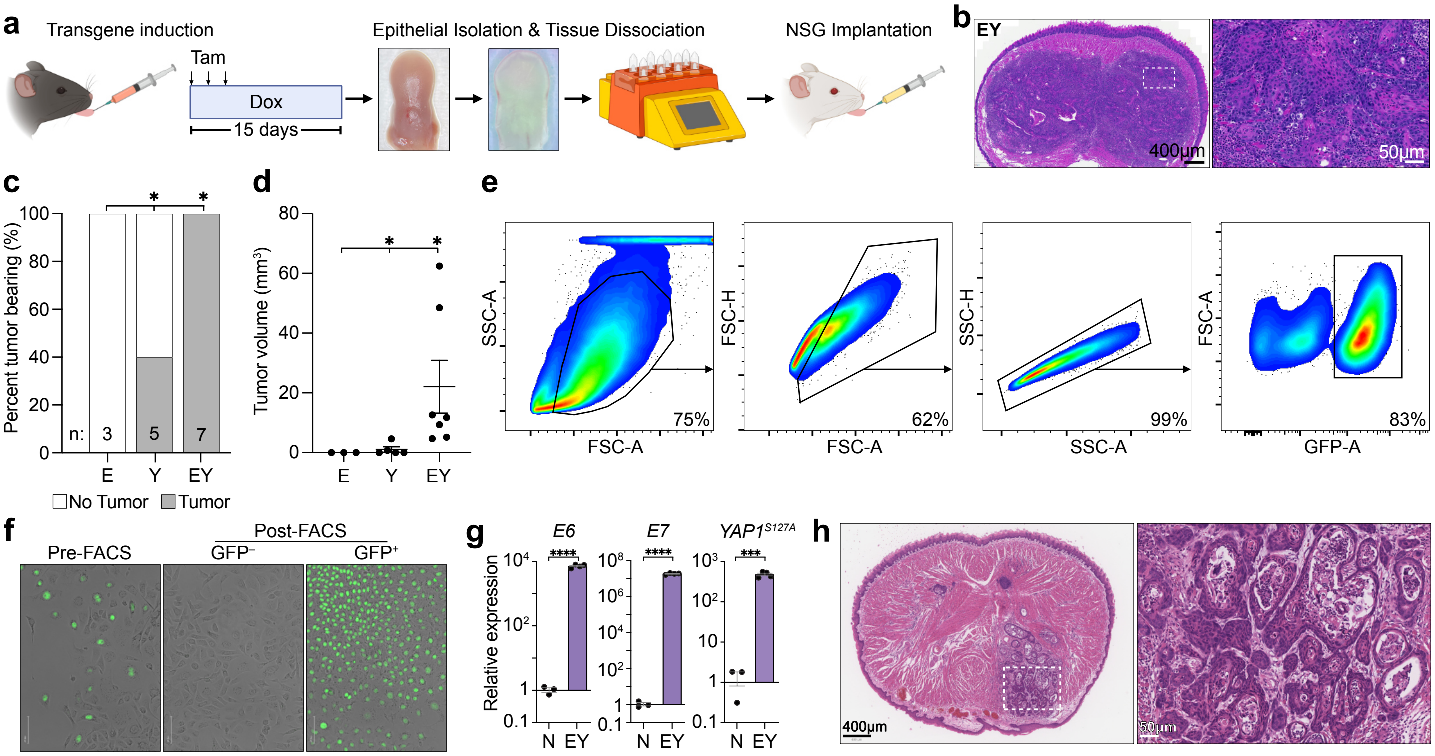

a
Transgene induction
Tam
Dox
15 days
Epithelial Isolation & Tissue Dissociation
NSG Implantation
b
EY
400μm
50μm
c
Percent tumor bearing (%)
n: 3 5 7
E Y EY
No Tumor
Tumor
d
Tumor volume (mm3)
E Y EY
e
SSC-A
FSC-A
75%
FSC-H
62%
SSC-H
SSC-A
99%
FSC-A
GFP-A
83%
f
Pre-FACS
Post-FACS
GFP−
GFP+
g
E6
E7
YAP1S127A
Relative expression
N EY
h
400μm
50μm

Supplementary Figure 2. YAP and E6-E7 activation in OEPCs induces tumor initiating cells (a) Experimental approach for the generation and orthotopic implantation of transgene-induced epithelial cell suspensions. Created in BioRender. Team, S. (2024) https://BioRender.com/t46s287 (b) Representative H&E stained section of an NSG mouse tongue after orthotopic implantation with transgene-induced EY epithelial cell suspension; scale bars 400μm (left), 50μm (right). (c) Implanted tumor outgrowth frequency; n, number of orthotopically implanted unique mouse tongue epithelia. Between group comparisons were made using Fisher's exact test with Bonferroni correction. (d) Tumor volumes 10 days after implantation. Mean with SEM is shown. ANOVA with Tukey correction for multiple comparisons used for between group comparisons. (e) Gating strategy for fluorescence-activated cell sorting (FACS)-based enrichment of H2B-GFP transgene positive cells derived from EY tongue epithelium based on GFP expression. Representative flow plots of n=9 samples. (f) Representative fluorescence microscopy of primary EY cell cultures pre- and post-FACS enrichment.(g) qRT-PCR quantification of HPV^{E6}, HPV^{E7}, and YAP^{S127A} expression in FACS-purified N and EY cell cultures. Barplots show mean and SEM. Mann-Whitney U test. (h) Representative H&E stained sections after implantation of 5,000 FACS-enriched EY cells in NSG mouse tongues; scale bars 400μm (left), 50μm (right). For all panels with asterisks denoting significance: *p<0.05, **p<0.01, ***p<0.001, ****p<0.0001. Source Data is available for panels c, d, and g. **Related to Fig. 1.**

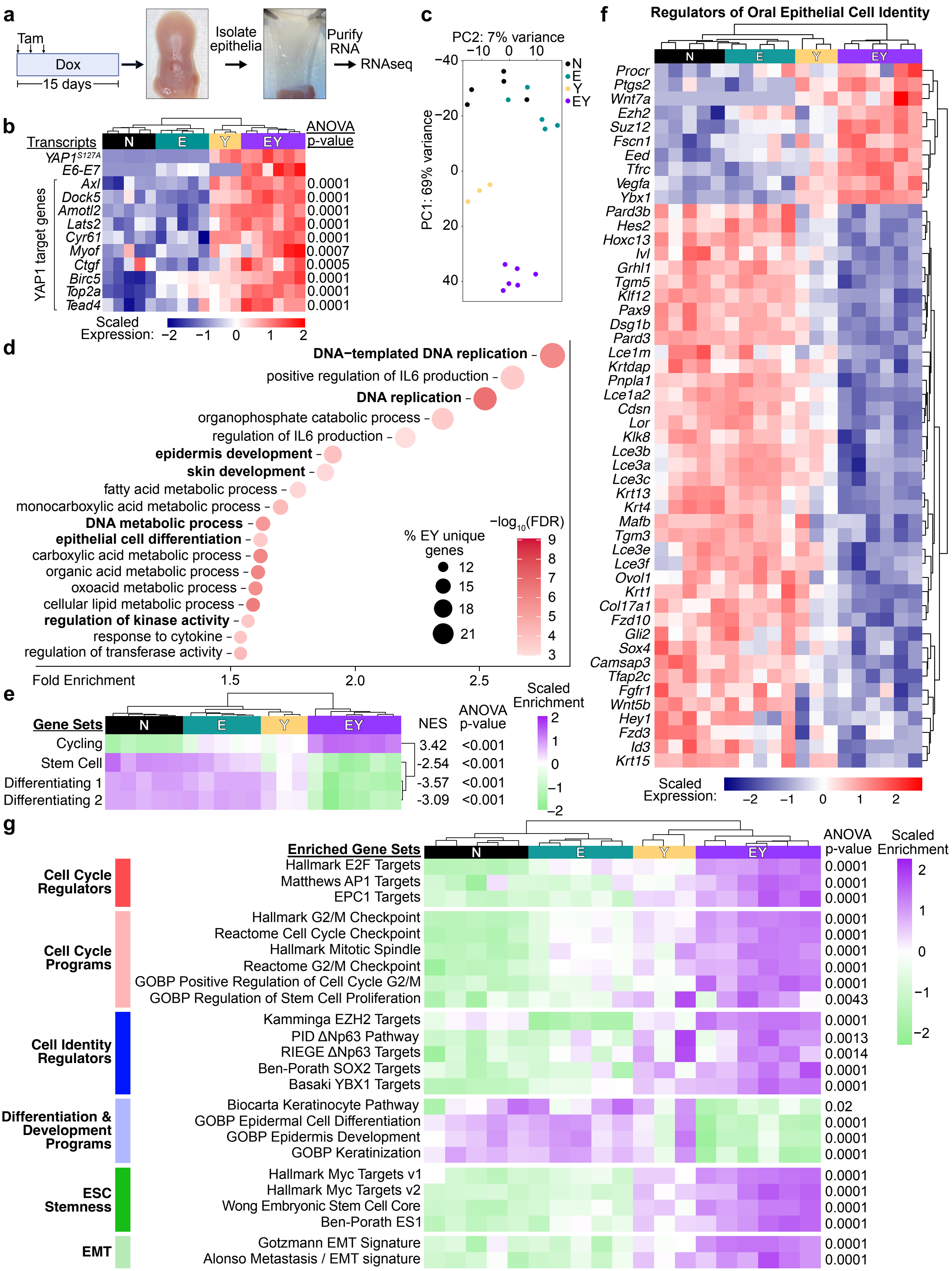

a
Tam
Dox
15 days
Isolate epithelia
Purify RNA
RNAseq
b
Transcripts
N
E
Y
EY
ANOVA p-value
YAP1 S127A
E6-E7
YAP1 target genes
Axl 0.0001
Dock5 0.0001
Amotl2 0.0001
Lats2 0.0001
Cyr61 0.0001
Myof 0.0007
Ctgf 0.0005
Birc5 0.0001
Top2a 0.0001
Tead4 0.0001
Scaled Expression: −2 −1 0 1 2
c
PC2: 7% variance
−10 0 10
PC1: 69% variance
−40 −20 0 20 40
N
E
Y
EY
d
DNA−templated DNA replication
positive regulation of IL6 production
DNA replication
organophosphate catabolic process
regulation of IL6 production
epidermis development
skin development
fatty acid metabolic process
monocarboxylic acid metabolic process
DNA metabolic process
epithelial cell differentiation
carboxylic acid metabolic process
organic acid metabolic process
oxoacid metabolic process
cellular lipid metabolic process
regulation of kinase activity
response to cytokine
regulation of transferase activity
% EY unique genes
12
15
18
21
−log10(FDR)
9 8 7 6 5 4 3
Fold Enrichment
1.5 2.0 2.5
e
Gene Sets
N
E
Y
EY
NES
ANOVA p-value
Scaled Enrichment
Cycling 3.42 <0.001
Stem Cell -2.54 <0.001
Differentiating 1 -3.57 <0.001
Differentiating 2 -3.09 <0.001
2 1 0 −1 −2
f
Regulators of Oral Epithelial Cell Identity
N
E
Y
EY
Procr
Ptgs2
Wnt7a
Ezh2
Suz12
Fscn1
Eed
Tfrc
Vegfa
Ybx1
Pard3b
Hes2
Hoxc13
Ivl
Grhl1
Tgm5
Klf12
Pax9
Dsg1b
Pard3
Lce1m
Krtdap
Pnpla1
Lce1a2
Cdsn
Lor
Klk8
Lce3b
Lce3a
Lce3c
Krt13
Krt4
Mafb
Tgm3
Lce3e
Lce3f
Ovol1
Krt1
Col17a1
Fzd10
Gli2
Sox4
Camsap3
Tfap2c
Fgfr1
Wnt5b
Hey1
Fzd3
Id3
Krt15
Scaled Expression: −2 −1 0 1 2
g
Enriched Gene Sets
N
E
Y
EY
ANOVA p-value
Scaled Enrichment
Cell Cycle Regulators
Hallmark E2F Targets 0.0001
Matthews AP1 Targets 0.0001
EPC1 Targets 0.0001
Cell Cycle Programs
Hallmark G2/M Checkpoint 0.0001
Reactome Cell Cycle Checkpoint 0.0001
Hallmark Mitotic Spindle 0.0001
Reactome G2/M Checkpoint 0.0001
GOBP Positive Regulation of Cell Cycle G2/M 0.0001
GOBP Regulation of Stem Cell Proliferation 0.0043
Cell Identity Regulators
Kamminga EZH2 Targets 0.0001
PID ΔNp63 Pathway 0.0013
RIEGE ΔNp63 Targets 0.0014
Ben-Porath SOX2 Targets 0.0001
Basaki YBX1 Targets 0.0001
Differentiation & Development Programs
Biocarta Keratinocyte Pathway 0.02
GOBP Epidermal Cell Differentiation 0.0001
GOBP Epidermis Development 0.0001
GOBP Keratinization 0.0001
ESC Stemness
Hallmark Myc Targets v1 0.0001
Hallmark Myc Targets v2 0.0001
Wong Embryonic Stem Cell Core 0.0001
Ben-Porath ES1 0.0001
EMT
Gotzmann EMT Signature 0.0001
Alonso Metastasis / EMT signature 0.0001
2 1 0 −1 −2

Supplementary Figure 3. Oncogenic transcriptional reprogramming defines YAP and E6-E7 activated epithelia

(a) Schematic demonstrating strategy for bulk RNAseq of microdissected tongue epithelia at 15 days post-transgene induction. (b) HPV^{E6E7}, YAP^{S127A}, and YAP target gene expression in transgenic epithelia transcriptomes. (c) Two-dimensional principal component analysis of individual N, E, Y, and EY transcriptome profiles. (d) Enriched cellular processes among EY unique DEGs by Gene Ontology using Panther. (e) Single sample GSEA showing enrichment or depletion of oral epithelial stem cell states including Cell Cycle Progression (G1/S, G2/M); Stem Cell; and Differentiation (Diff 1 and Diff 2). These gene signatures were derived from cluster defining genes from Jones et al. See Supplementary Data 3 for gene set details. (f) Relative expression of EY-unique DEGs regulating oral epithelial cell identity. (g) Single sample GSEA for all transgenic conditions for gene signatures related to cell cycle, differentiation, stemness, and EMT. See Supplementary Data 3 for gene set details. For all data presented in this figure: N=5, E=5, Y=3, and EY=6 biological replicates. Each row in panels b, e, f, and g was analyzed by ANOVA with Tukey correction for multiple comparisons: *$p<0.05$, **$p<0.01$, ***$p<0.001$, ****$p<0.0001$.

Related to Fig. 2.

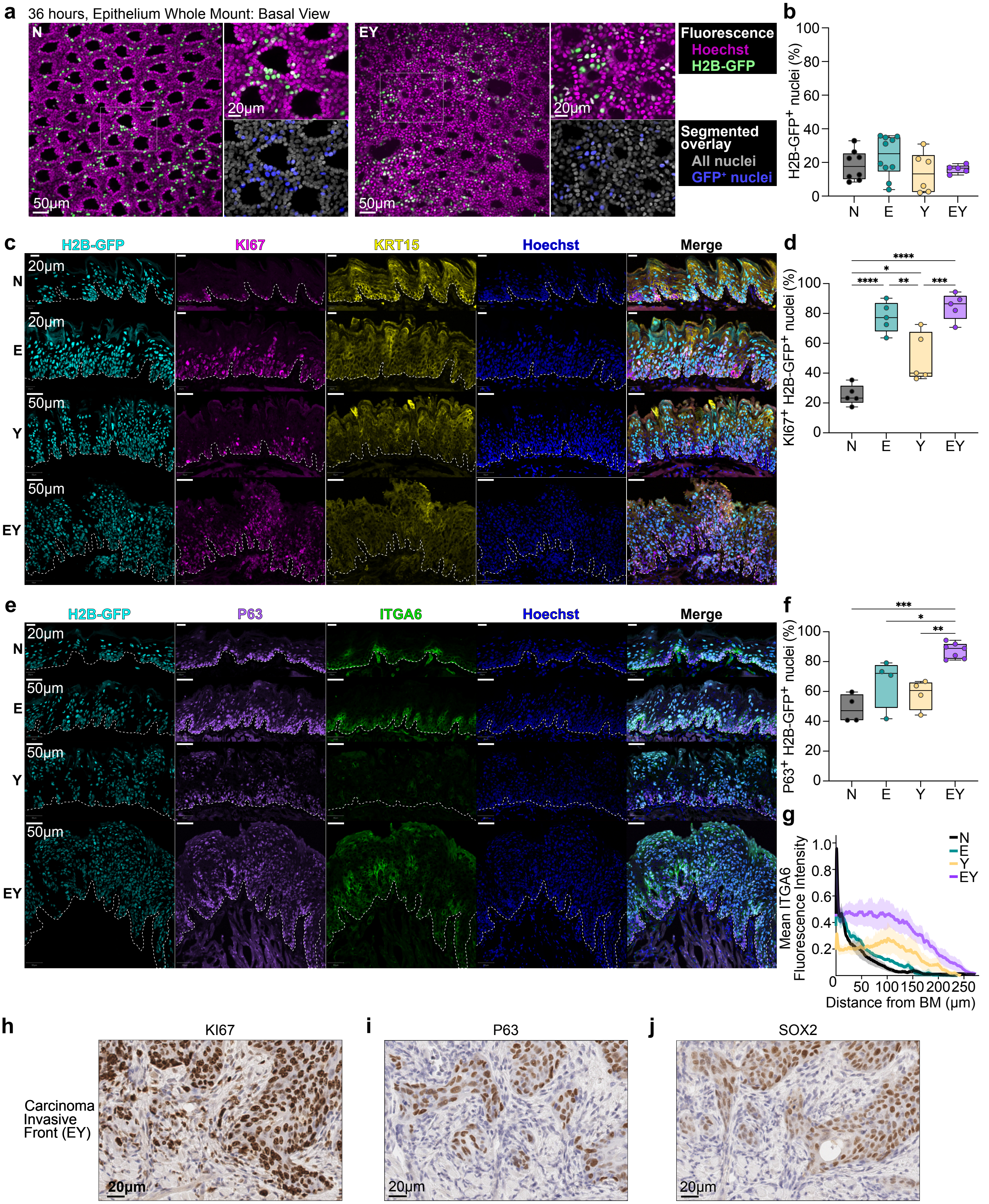

a
36 hours, Epithelium Whole Mount: Basal View
N
EY
20µm
50µm
Fluorescence
Hoechst
H2B-GFP
Segmented overlay
All nuclei
GFP⁺ nuclei
b
H2B-GFP⁺ nuclei (%)
N
E
Y
EY
c
H2B-GFP
KI67
KRT15
Hoechst
Merge
d
KI67⁺ H2B-GFP⁺ nuclei (%)
e
P63
ITGA6
f
P63⁺ H2B-GFP⁺ nuclei (%)
g
Mean ITGA6 Fluorescence Intensity
Distance from BM (µm)
h
KI67
i
P63
j
SOX2
Carcinoma Invasive Front (EY)

Supplementary Figure 4. Invasive carcinoma is preceded by the expansion of a proliferative, stem-like cell population

(a-b) Lineage tracing by fluorescent microscopy using the *H2B-GFP* reporter transgene to track and quantify GFP^+ nuclei. (a) Representative fluorescence images and (b) percent basal H2B-GFP^+ nuclei in tongue epithelial basal layer whole mounts 36 hours after transgene induction; scale bars 20μm (left), 50μm (right). (c) Representative immunofluorescence images of H2B-GFP, KI67, and KRT15 expression in tongue epithelia 15 days after transgene induction. White dashed line represents the basement membrane; scale bars 20μm for N and E, 50μm for Y and EY. (d) Related to c. Percent $KI67^+GFP^+$ nuclei in tongue epithelia. (e) Representative immunofluorescence images of H2B-GFP, P63, and ITGA6 expression in tongue epithelia 15 days after transgene induction. White dashed line represents the basement membrane; scale bars 20μm for N, 50μm for E, Y and EY. (f) Related to e. Percent $P63^+GFP^+$ nuclei in tongue epithelia. (g) Related to e. Quantitative spatial analysis showing normalized fluorescent intensity of ITGA6 signal from basement membrane to epidermal surface. Probability cloud shows 95% confidence interval. (h-j) Magnified images of cells at the invasive front from Fig. 2m demonstrating (h) $KI67^+$ nuclei in Fig. 2i, (i) $P63^+$ nuclei in Fig. 2k, (j) $SOX2^+$ nuclei in Fig. 2m; scale bars all 20μm. Panels b, d, and f were analyzed by ANOVA with Tukey correction for multiple comparisons: *p<0.05, **p<0.01, ***p<0.001, ****p<0.0001. Boxplots show median, interquartile range (IQR), and range.

Related to Fig. 2.

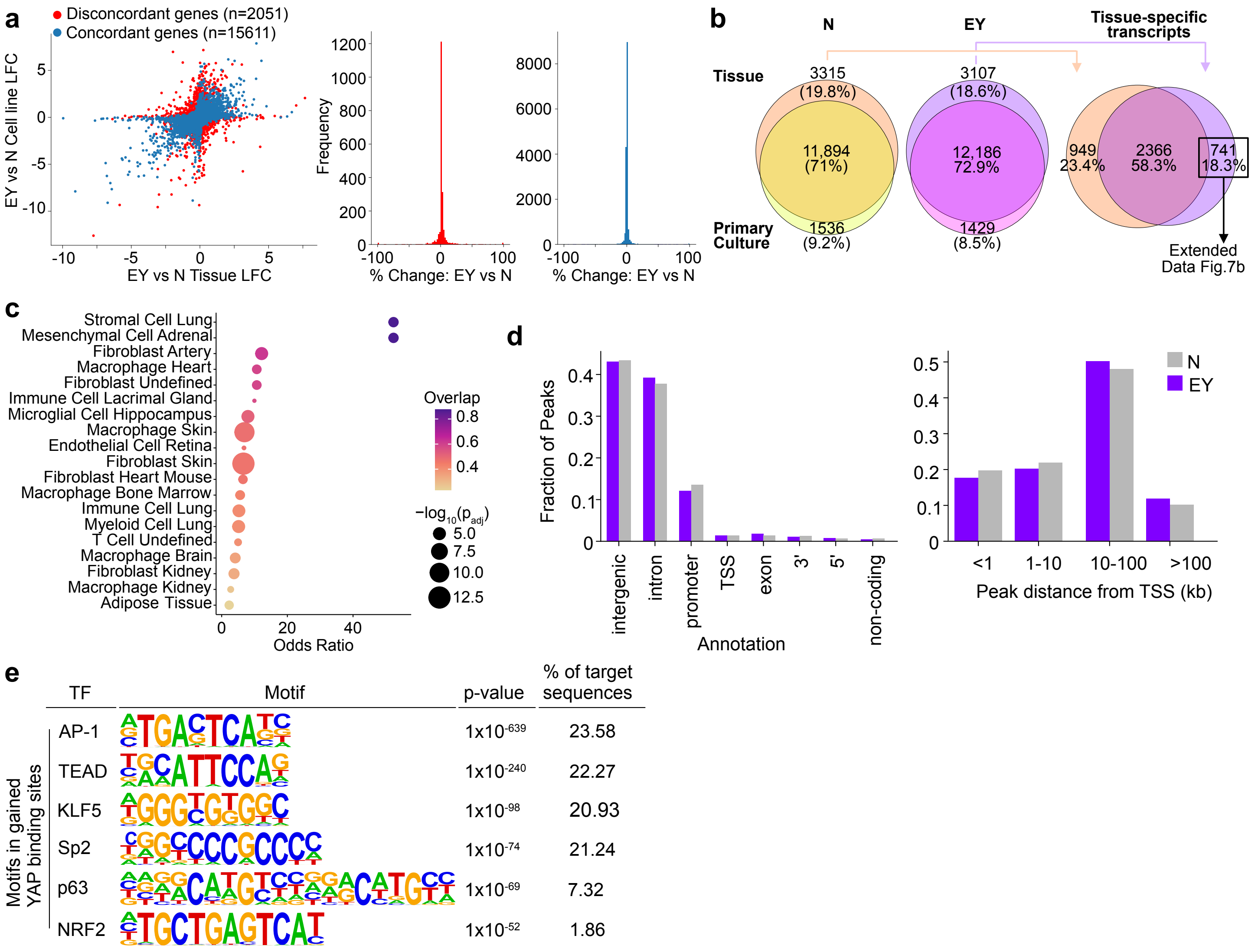

a
Disconcordant genes (n=2051)
Concordant genes (n=15611)
EY vs N Cell line LFC
EY vs N Tissue LFC
Frequency
% Change: EY vs N
b
N
EY
Tissue-specific transcripts
Tissue
Primary Culture
3315 (19.8%)
11,894 (71%)
1536 (9.2%)
3107 (18.6%)
12,186 72.9%
1429 (8.5%)
949 23.4%
2366 58.3%
741 18.3%
Extended Data Fig.7b
c
Stromal Cell Lung
Mesenchymal Cell Adrenal
Fibroblast Artery
Macrophage Heart
Fibroblast Undefined
Immune Cell Lacrimal Gland
Microglial Cell Hippocampus
Macrophage Skin
Endothelial Cell Retina
Fibroblast Skin
Fibroblast Heart Mouse
Macrophage Bone Marrow
Immune Cell Lung
Myeloid Cell Lung
T Cell Undefined
Macrophage Brain
Fibroblast Kidney
Macrophage Kidney
Adipose Tissue
Odds Ratio
Overlap
−log10(padj)
d
Fraction of Peaks
intergenic
intron
promoter
TSS
exon
3'
5'
non-coding
Annotation
N
EY
<1
1-10
10-100
>100
Peak distance from TSS (kb)
e
TF
Motif
p-value
% of target sequences
Motifs in gained YAP binding sites
AP-1 1x10-639 23.58
TEAD 1x10-240 22.27
KLF5 1x10-98 20.93
Sp2 1x10-74 21.24
p63 1x10-69 7.32
NRF2 1x10-52 1.86

Supplementary Figure 5. Epigenetic reprogramming in YAP and E6-E7 activated epithelia and EY cell lines

(a) Left: DESeq interaction model comparing genes enriched in EY compared to N tissue versus in EY compared to N primary cells. Discordant genes between epithelial tissue and cell lines are shown in red, and concordant genes (88%) in blue. Concordance threshold based on log fold difference, $p > 0.05$. Right: Frequency of genes with an observed difference (%) in EY versus N across EY and N tissues and cell lines. (b) Left two Venn diagrams: overlap of transcripts detected in N (left) or EY (middle) epithelial tissue (top circles) versus primary cell culture (bottom circles). Right Venn diagram: overlap of tissue-specific N or EY transcripts shows 741 transcripts unique to EY epithelial tissue. (c) Enrichr analysis of the 2366 tissue-specific transcripts detected in both N and EY tissue found in the Cell Marker 2024 library. (d) Left: Fraction of YAP CUT&Tag peaks that localize to genomic region annotations defined using HOMER. Right: Absolute distance of YAP CUT&Tag peaks to the nearest transcription start site (TSS). (e) Top transcription factor binding motifs enriched in the EYgained YAP CUT&Tag binding sites. P values were calculated using HOMER. YAP CUT&Tag: N=2, EY=4 biological replicates; RNAseq: N=4, EY=6 biological replicates.

Related to Fig. 3.

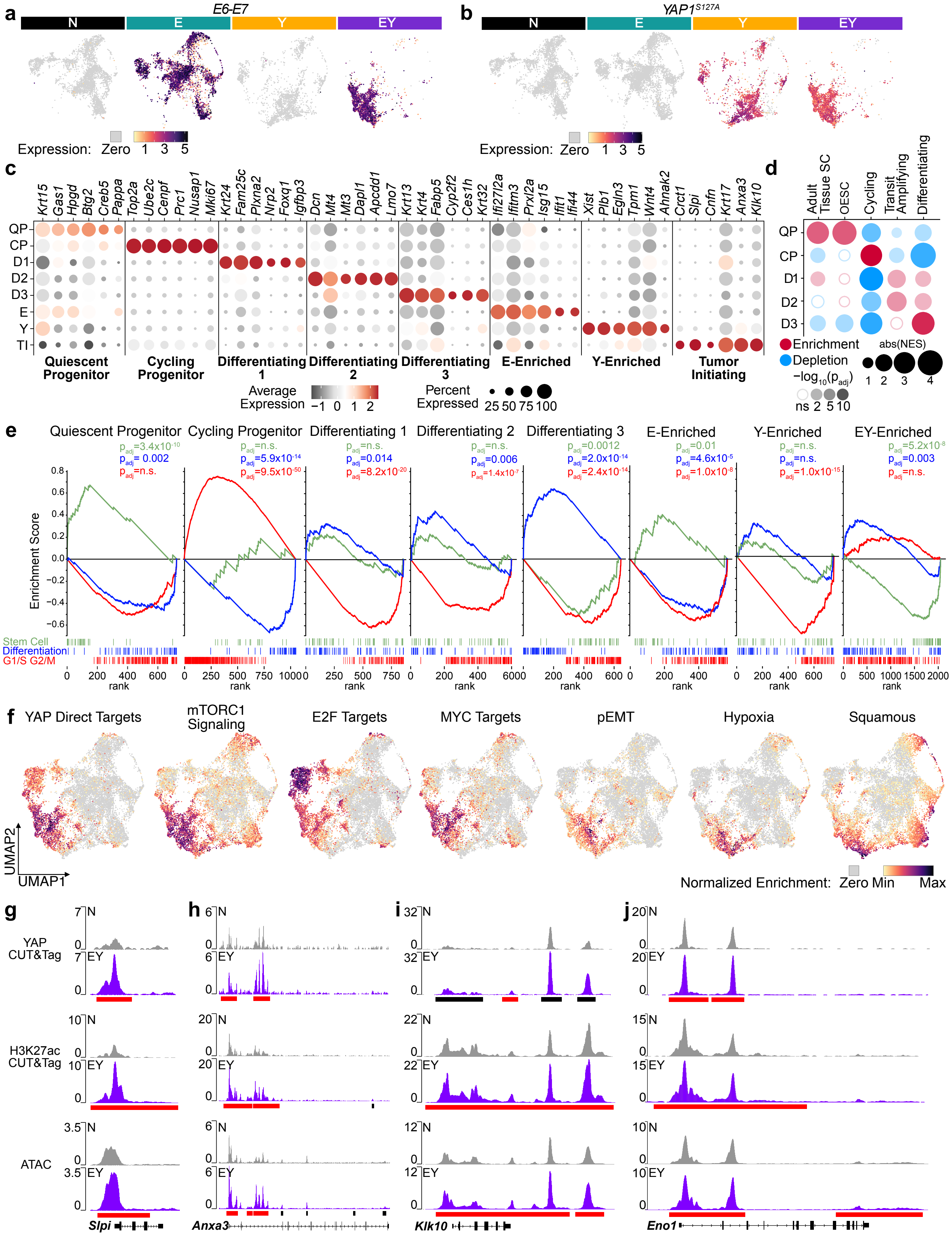
a
E6-E7
N
E
Y
EY
Expression: Zero 1 3 5
b
YAP1S127A
N
E
Y
EY
Expression: Zero 1 3 5
c
Krt15 Gas1 Hpgd Btg2 Creb5 Pappa Top2a Ube2c Cenpf Prc1 Nusap1 Mki67 Krt24 Fam25c Plxna2 Nrp2 Foxq1 Igfbp3 Dcn Mt4 Mt3 Dapl1 Apcdd1 Lmo7 Krt13 Krt4 Fabp5 Cyp2f2 Ces1h Krt32 Ifi27l2a Ifitm3 Prxl2a Isg15 Ifit1 Ifi44 Xist Plb1 Egln3 Tpm1 Wnt4 Ahnak2 Crct1 Slpi Cnfn Krt17 Anxa3 Klk10
QP
CP
D1
D2
D3
E
Y
TI
Quiescent Progenitor
Cycling Progenitor
Differentiating 1
Differentiating 2
Differentiating 3
E-Enriched
Y-Enriched
Tumor Initiating
Average Expression -1 0 1 2
Percent Expressed 25 50 75 100
d
Adult Tissue SC
OESC
Cycling
Transit Amplifying
Differentiating
QP
CP
D1
D2
D3
Enrichment
Depletion
−log10(padj)
ns 2 5 10
abs(NES)
1 2 3 4
e
Quiescent Progenitor
Cycling Progenitor
Differentiating 1
Differentiating 2
Differentiating 3
E-Enriched
Y-Enriched
EY-Enriched
padj=3.4x10-10
padj= 0.002
padj=n.s.
padj=n.s.
padj=5.9x10-14
padj=9.5x10-50
padj=n.s.
padj=0.014
padj=8.2x10-20
padj=n.s.
padj=0.006
padj=1.4x10-7
padj=0.0012
padj=2.0x10-14
padj=2.4x10-14
padj=0.01
padj=4.6x10-5
padj=1.0x10-8
padj=n.s.
padj=n.s.
padj=1.0x10-15
padj=5.2x10-8
padj=0.003
padj=n.s.
Enrichment Score
Stem Cell
Differentiation
G1/S G2/M
rank
f
YAP Direct Targets
mTORC1 Signaling
E2F Targets
MYC Targets
pEMT
Hypoxia
Squamous
UMAP2
UMAP1
Normalized Enrichment: Zero Min Max
g
h
i
j
YAP CUT&Tag
H3K27ac CUT&Tag
ATAC
N
EY
Slpi
Anxa3
Klk10
Eno1

Supplementary Figure 6. Single cell analysis of E6-E7 and YAP activated epithelial cells Feature plots of (a) *E6-E7* and (b) *YAP1^{S127A}* transgene expression stratified by genotype; N=3,783, E=4,529, Y=2,311, EY=2,148 cells. (c) Expression of the top 6 cluster defining genes among epithelial cell clusters. Dot size denotes the percentage of cells within a cluster expressing each transcript and the color indicates average gene expression across all cells in each cluster. (d) GSEA of physiologic cell states across epithelial cell clusters. Circle color indicates enrichment (red) or depletion (blue). Circle size encodes the absolute value (abs) of the normalized enrichment score (NES). Circle opacity represents -log10 of the adjusted p-value (padj); circles are hollow if padj>0.05. For gene set details, please see Supplementary Data 3. (e) GSEA enrichment plots for the Jones Stem Cell, Jones Differentiation, and Jones G1/S G2/M physiologic OEPC gene sets across epithelial cell clusters. (f) Feature plots showing expression of YAP Direct Target, mTORC1 Signaling, E2F Targets, MYC Targets, and the Barkley et al. recurring cancer cell state gene sets: partial epithelial to mesenchymal transition (pEMT), hypoxia, and squamousdifferentiation, n=12,771 cells in each UMAP (n=2 mice per group). IGV tracks of YAP CUT&Tag, H3K27ac CUT&Tag, and ATACseq peaks at the (g) Slpi, (h) Anxa3, (i) Klk10, and (j) Eno1 gene loci. For panels g-j: black bars indicate significant peaks. Red bars indicate EY-gained peaks. YAP and H3K27ac CUT&Tag: N=2, EY=4 biological replicates; ATACseq: 4 biological replicates per condition.
Related to Fig. 4.

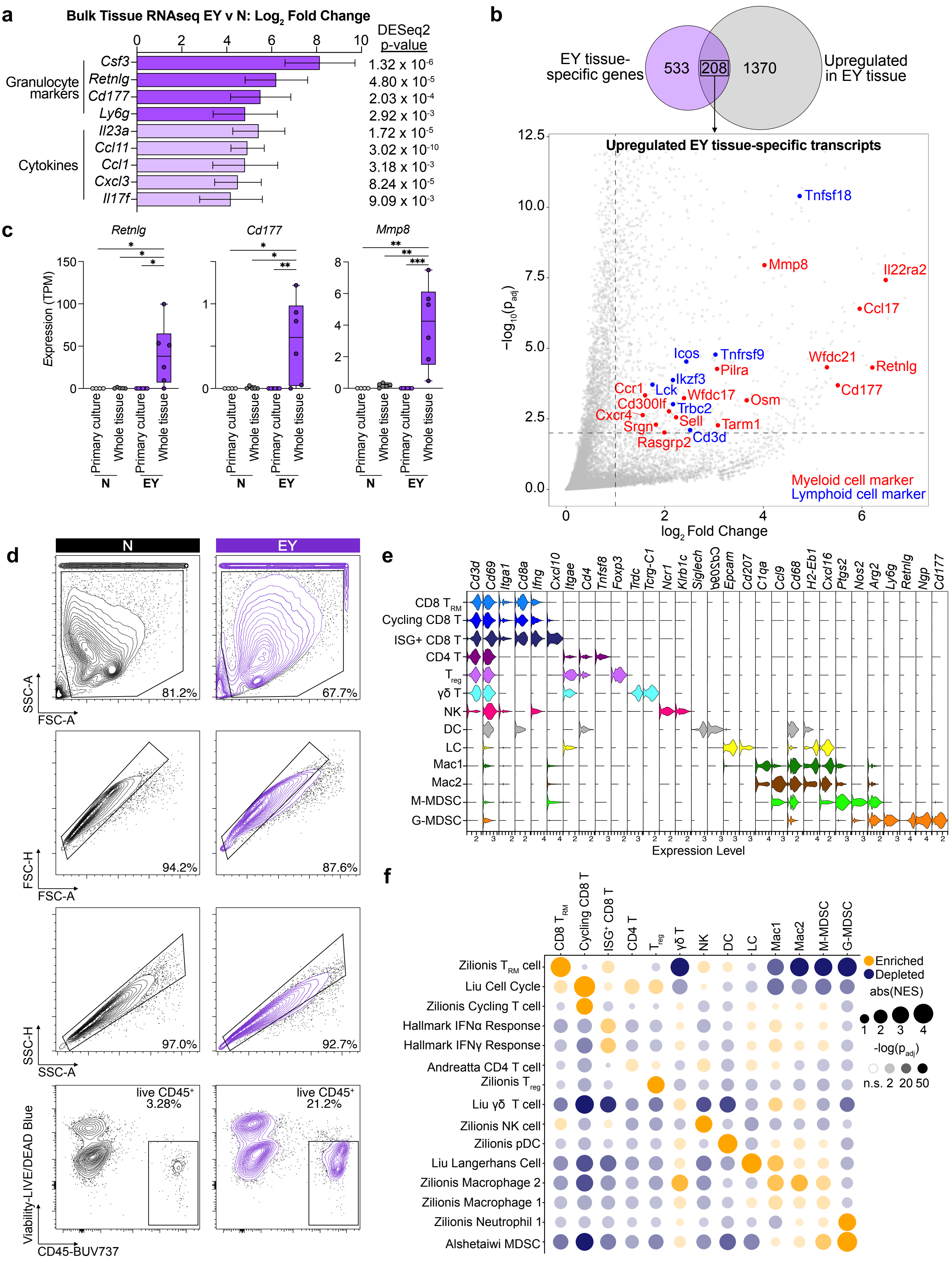
a
Bulk Tissue RNAseq EY v N: Log2 Fold Change
0 2 4 6 8 10
DESeq2 p-value
Granulocyte markers
Csf3
Retnlg
Cd177
Ly6g
Cytokines
Il23a
Ccl11
Ccl1
Cxcl3
Il17f
1.32 x 10-6
4.80 x 10-5
2.03 x 10-4
2.92 x 10-3
1.72 x 10-5
3.02 x 10-10
3.18 x 10-3
8.24 x 10-5
9.09 x 10-3
b
EY tissue-specific genes
533
208
1370
Upregulated in EY tissue
Upregulated EY tissue-specific transcripts
-log10(padj)
log2 Fold Change
Tnfsf18
Mmp8
Il22ra2
Ccl17
Wfdc21
Retnlg
Cd177
Icos
Tnfrsf9
Pilra
Ikzf3
Ccr1
Lck
Wfdc17
Cd300lf
Trbc2
Osm
Cxcr4
Sell
Srgn
Tarm1
Rasgrp2
Cd3d
Myeloid cell marker
Lymphoid cell marker
c
Retnlg
Cd177
Mmp8
Expression (TPM)
Primary culture
Whole tissue
N
EY
d
N
EY
SSC-A
FSC-A
81.2%
67.7%
FSC-H
94.2%
87.6%
SSC-H
SSC-A
97.0%
92.7%
live CD45+ 3.28%
live CD45+ 21.2%
Viability-LIVE/DEAD Blue
CD45-BUV737
e
Cd3d Cd69 Itga1 Cd8a Ifng Cxcl10 Itgae Cd4 Tnfsf8 Foxp3 Trdc Tcrg-C1 Ncr1 Klrb1c Siglech Cd300c Epcam Cd207 C1qa Ccl9 Cd68 H2-Eb1 Cxcl16 Ptgs2 Nos2 Arg2 Ly6g Retnlg Ngp Cd177
CD8 TRM
Cycling CD8 T
ISG+ CD8 T
CD4 T
Treg
γδ T
NK
DC
LC
Mac1
Mac2
M-MDSC
G-MDSC
Expression Level
f
CD8 TRM
Cycling CD8 T
ISG+ CD8 T
CD4 T
Treg
γδ T
NK
DC
LC
Mac1
Mac2
M-MDSC
G-MDSC
Zilionis TRM cell
Liu Cell Cycle
Zilionis Cycling T cell
Hallmark IFNα Response
Hallmark IFNγ Response
Andreatta CD4 T cell
Zilionis Treg
Liu γδ T cell
Zilionis NK cell
Zilionis pDC
Liu Langerhans Cell
Zilionis Macrophage 2
Zilionis Macrophage 1
Zilionis Neutrophil 1
Alshetaiwi MDSC
Enriched
Depleted
abs(NES)
1 2 3 4
-log(padj)
n.s. 2 20 50

Supplementary Figure 7. Single cell analysis of E6-E7 and YAP polarized epithelial immune infiltrate

(a) RNAseq data from bulk epithelia for granulocyte markers and cytokines among EY DEGs, generated using DESeq2; means with SEM are shown; N=5, EY=6 biological replicates of bulk tissue RNAseq. (b) Top: Venn diagram showing overlap of EY tissue-specific and EY upregulated genes. Bottom: Volcano plot of epithelial bulk RNAseq data comparing EY vs N highlighting EY upregulated tissue-specific genes. Myeloid cell associated genes enriched in EY epithelial tissue are shown in red and lymphoid cell associated genes enriched in EY epithelial tissue are shown in blue; N=5, EY=6 of bulk tissue RNAseq. (c) Bulk RNAseq expression in transcripts per million (TPM) of the EY upregulated tissue-specific myeloid cell markers, *Retnlg*, *Cd177*, and *Mmp8*, across N and EY epithelial tissue (N=5, EY=6) and primary cells (N=4, EY=6). Boxplots show median, interquartile range, and range. One-way ANOVA with Tukey correction for multiple comparisons. (d) Gating strategy used to identify viable $CD45^+$ cells in transgene-induced tongue epithelia; related to Figure 5a; from days 0-15: N=5, 3, 10, and 21 mice; E=5, 5, 11, and 21 mice; Y=4, 3, 8, and 17 mice; EY=3, 1, 9, and 16 mice. (e) Expression of select cell type defining genes among scRNAseq immune clusters. (f) Dot Plot demonstrating GSEA of published cell-type defining gene lists across immune cell types. Dot color indicates enrichment (Orange) or depletion (Blue). Dot size represents the absolute value of the normalized enrichment score (NES). Dot opacity represents $-\log_{10}$ of the adjusted p-value. For gene set details, please see Supplementary Data 3.

Related to Fig. 5.

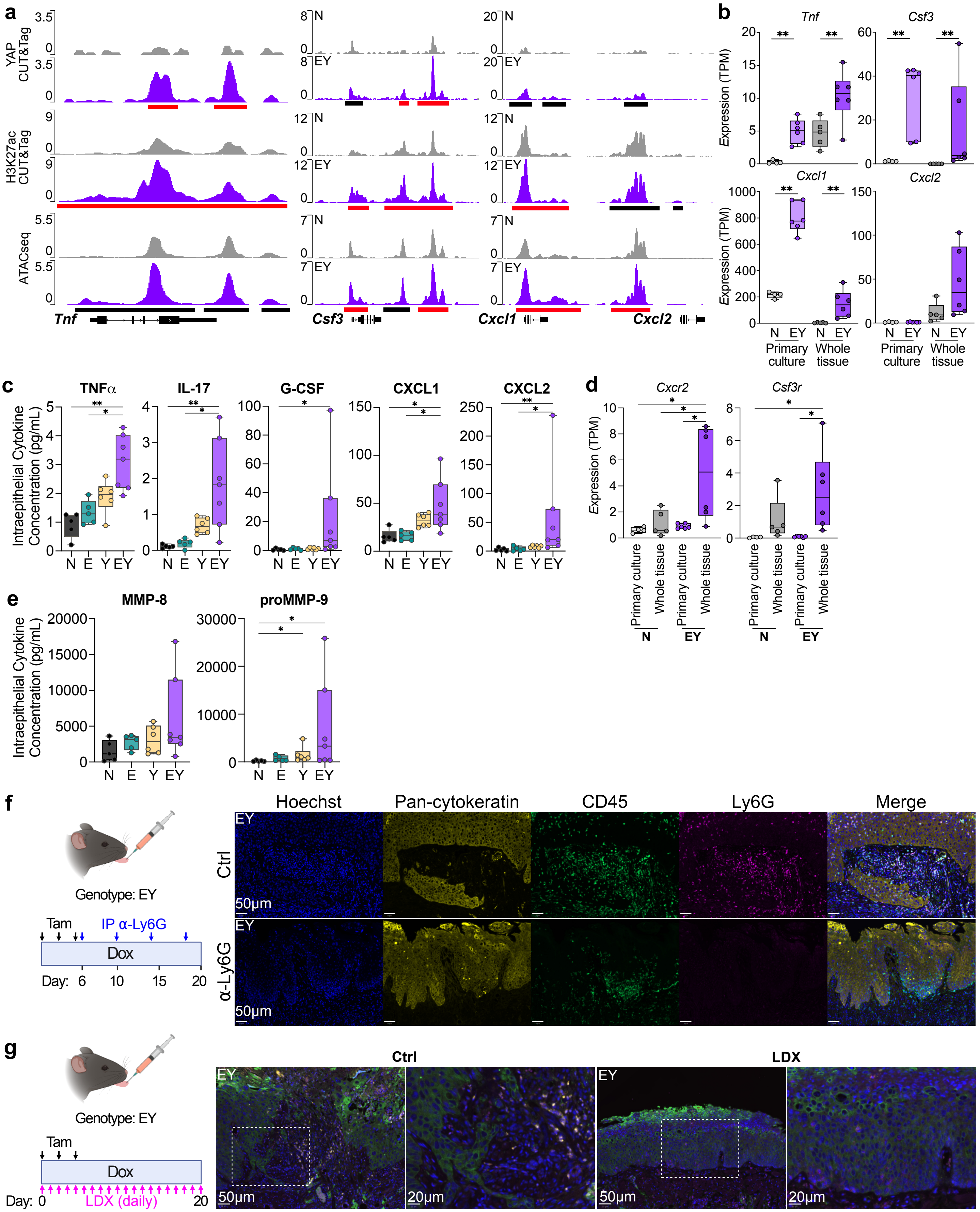
a
YAP CUT&Tag
H3K27ac CUT&Tag
ATACseq
N
EY
Tnf
Csf3
Cxcl1
Cxcl2
b
Tnf
Csf3
Cxcl1
Cxcl2
Expression (TPM)
N EY N EY
Primary culture
Whole tissue
c
TNFα
IL-17
G-CSF
CXCL1
CXCL2
Intraepithelial Cytokine Concentration (pg/mL)
N E Y EY
d
Cxcr2
Csf3r
Expression (TPM)
Primary culture
Whole tissue
N
EY
e
MMP-8
proMMP-9
Intraepithelial Cytokine Concentration (pg/mL)
N E Y EY
f
Genotype: EY
Tam
IP α-Ly6G
Dox
Day: 6 10 15 20
Hoechst
Pan-cytokeratin
CD45
Ly6G
Merge
Ctrl
α-Ly6G
EY
50μm
g
Genotype: EY
Tam
Dox
Day: 0 20
LDX (daily)
Ctrl
LDX
EY
50μm
20μm

Supplementary Figure 8.
(a) IGV tracks of YAP CUT&Tag, H3K27ac CUT&Tag, and ATACseq peaks at the *Tnf, Csf3, Cxcl1, Cxcl2* gene loci. Black bars indicate significant peaks. Red bars indicate EY-gained peaks. YAP and H3K27ac CUT&Tag: N=2, EY=4 biological replicates; ATACseq: 4 biological replicates per condition. (b) Bulk RNAseq expression in TPM of *Tnf, Csf3, Cxcl1, Cxcl2* across N and EY epithelial tissue (N=5, EY=6) and primary cells (N=4, EY=6). (c) G-CSF, IL-17, TNFα, CXCL1, and CXCL2 protein abundance by cytokine array in epithelial lysates from N, E, Y, and EY mice 15 days after transgene induction; N=5, E=5, Y=6, EY=7 lysates from mouse tongue epithelia. (d) Bulk RNAseq expression in TPM of *Cxcr2, Csf3r* across N and EY epithelial tissue (N=5, EY=6) and primary cells (N=4, EY=6). (e) MMP8 and pro-MMP9 protein abundance by cytokine array in epithelial lysates from N, E, Y, and EY mice 15 days after transgene induction; N=5, E=5, Y=6, EY=7 lysates from mouse tongue epithelia. (f) Left: Experimental approach for depletion of $LY6G^+$ G-MDSCs in transgene induced EY mice. Right: Representative images of $CD45^+$ and $Ly6G^+$ immune infiltrates in EY mouse tongue epithelia 20 days after transgene induction after treatment with vehicle (top, n=11) or anti-Ly6G depleting antibody (bottom, n=9); scale bars all 50μm (g) Left: Experimental approach for treatment with CXCR1/2 dual inhibitor ladarixin in transgene induced EY mice. Right: Representative images of $CD45^+$ and $Ly6G^+$ immune infiltrates in EY mouse tongue epithelia 20 days after transgene induction after treatment with vehicle (Left, n=23) or ladarixin (Right, n=12); scale bars 50μm (main image) and 20μm (inset). Panels b-e were analyzed by ANOVA with Tukey correction for multiple comparisons. Boxplots show median, interquartile range (IQR), and range. $*p<0.05$, $**p<0.01$. Panels f,g were created in BioRender. Team, S. (2024) https://BioRender.com/u67h199.
Related to Fig. 5.

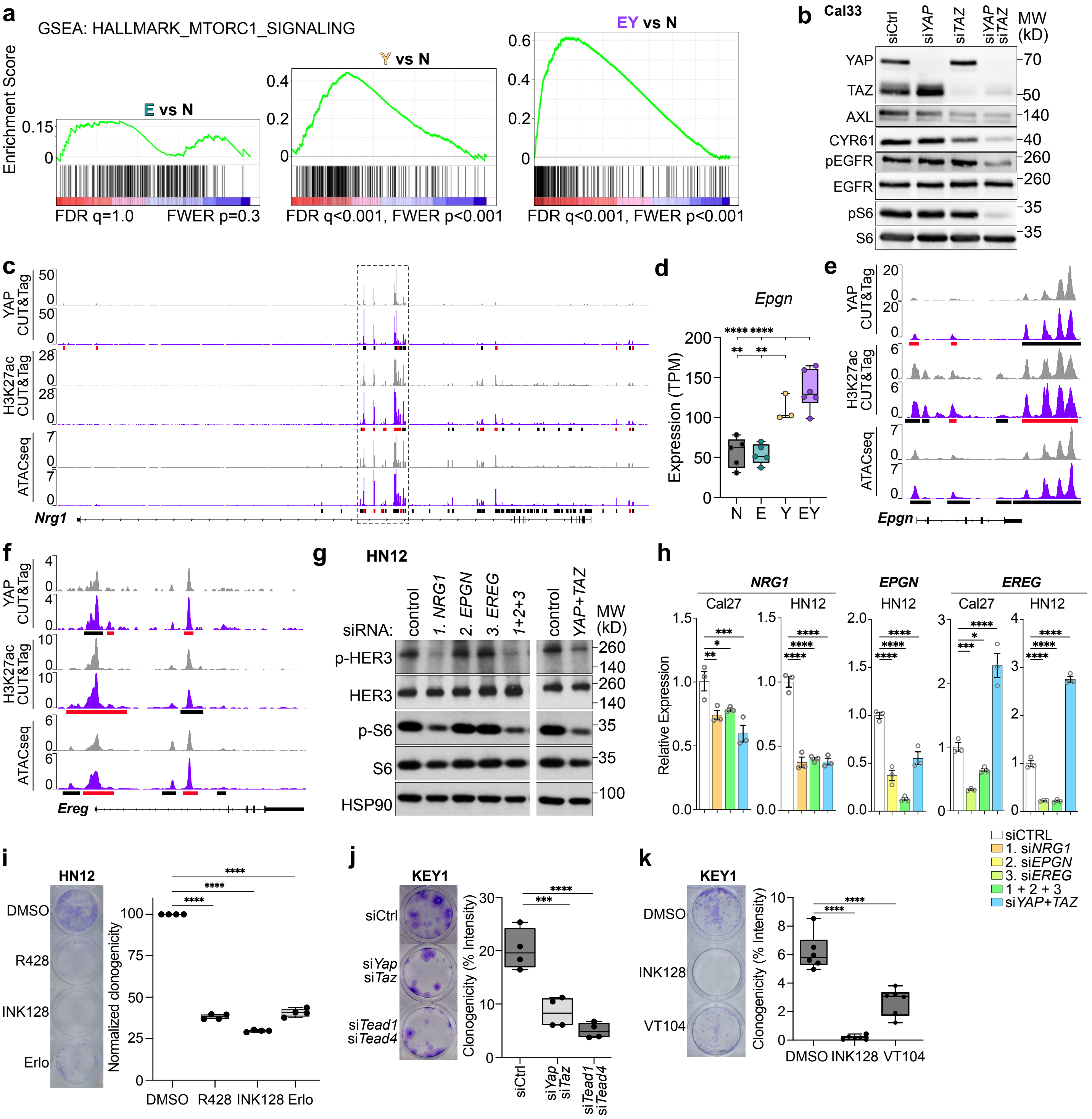
a
GSEA: HALLMARK_MTORC1_SIGNALING
Enrichment Score
E vs N
FDR q=1.0
FWER p=0.3
Y vs N
FDR q<0.001, FWER p<0.001
EY vs N
FDR q<0.001, FWER p<0.001
b
Cal33
siCtrl
siYAP
siTAZ
siYAP siTAZ
MW (kD)
YAP
TAZ
AXL
CYR61
pEGFR
EGFR
pS6
S6
c
YAP CUT&Tag
H3K27ac CUT&Tag
ATACseq
Nrg1
d
Epgn
Expression (TPM)
N
E
Y
EY
e
YAP CUT&Tag
H3K27ac CUT&Tag
ATACseq
Epgn
f
YAP CUT&Tag
H3K27ac CUT&Tag
ATACseq
Ereg
g
HN12
siRNA:
control
1. NRG1
2. EPGN
3. EREG
1+2+3
control
YAP+TAZ
MW (kD)
p-HER3
HER3
p-S6
S6
HSP90
h
NRG1
EPGN
EREG
Cal27
HN12
Relative Expression
siCTRL
1. siNRG1
2. siEPGN
3. siEREG
1 + 2 + 3
siYAP+TAZ
i
HN12
DMSO
R428
INK128
Erlo
Normalized clonogenicity
j
KEY1
siCtrl
siYap siTaz
siTead1 siTead4
Clonogenicity (% Intensity)
k
KEY1
DMSO
INK128
VT104
Clonogenicity (% Intensity)

Supplementary Figure 9. YAP-mediated transcriptional activation of mTOR signaling
(a) GSEA enrichment plots for the HALLMARK_MTORC1_SIGNALING gene set for E-, Y-, and EY differentially expressed genes compared to N; N=5, E=5, Y=3, EY=6 biological replicates (b) Western blot showing effect of siRNA-mediated knockdown of YAP, TAZ, and AXL on YAP target gene (AXL, CYR61) and phospho-EGFR and phospho-S6 ribosomal protein abundance in Cal33 cells. (c) IGV tracks of YAP CUT&Tag, H3K27ac CUT&Tag, and ATACseq peaks at the *Nrg1* gene locus. Dotted line box indicates segment displayed in Fig. 6f. (d) *Epgn* expression in transgenic tongue epithelia by RNAseq. IGV tracks of YAP CUT&Tag, H3K27ac CUT&Tag, and ATACseq peaks at (e) *Epgn* and (f) *Ereg* gene loci. YAP and H3K27ac CUT&Tag: N=2, EY=4 biological replicates; ATACseq: 4 biological replicates per condition. (g) Phospho- and total HER3 and S6 expression in HN12 whole cell lysate following siRNA-mediated knockdown of *NRG1*, *EREG*, *EPGN*. (h) *NRG1*, *EREG*, *EPGN* transcript expression in Cal27 and HN12 cells following siRNA-mediated knockdown of *NRG1*, *EREG*, *EPGN, YAP, or TAZ*; 3 biological replicates per condition. Mean with standard error of the mean; ANOVA with Tukey correction for multiple comparisons. (i) Representative wells (left) and quantification (right) of clonogenic assays in HN12 cells treated with DMSO, R428, INK128, or Erlotinib (Erlo); 4 biological replicates per condition. (j) Representative wells (left) and quantification (right) of clonogenic assays in EY primary cells treated with siRNA targeting, *YAP+TAZ* or *TEAD1+TEAD4*; 4 biological replicates per condition. (k) Representative wells (left) and quantification (right) of clonogenic assays in EY primary cells treated with mTOR inhibitor rapamycin or YAP-TEAD inhibitor VT104; 6 biological replicates per condition. For panels c, e , f black bars indicate significant peaks. Red bars indicate EY-gained peaks. For panels d, i, j, k boxplots show median, interquartile range (IQR), and range; ANOVA with Tukey correction for multiple comparisons. *$p<0.05$, **$p<0.01$, ***$p<0.001$, ****$p<0.0001$.
Related to Fig. 6.

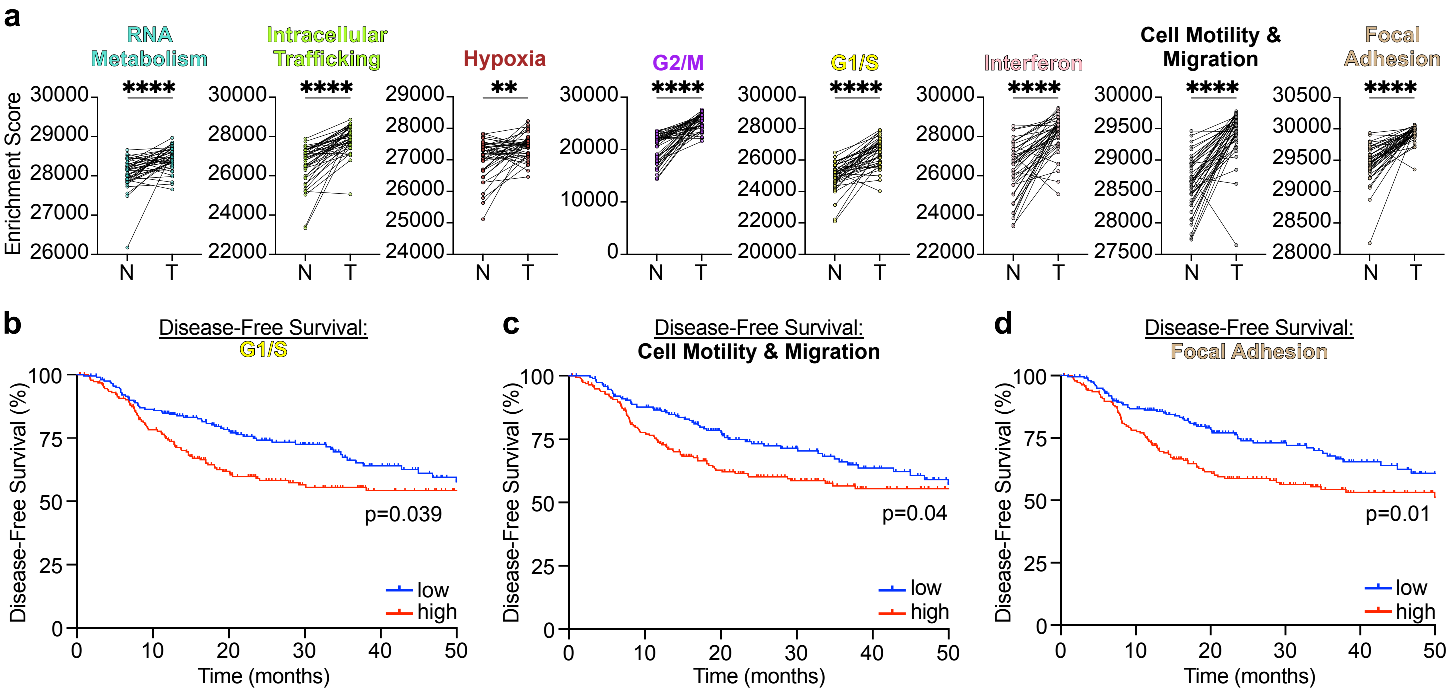

a
RNA Metabolism
Intracellular Trafficking
Hypoxia
G2/M
G1/S
Interferon
Cell Motility & Migration
Focal Adhesion
****
**
Enrichment Score
N
T
b
Disease-Free Survival: G1/S
p=0.039
c
Disease-Free Survival: Cell Motility & Migration
p=0.04
d
Disease-Free Survival: Focal Adhesion
p=0.01
Disease-Free Survival (%)
Time (months)
low
high

Supplementary Figure 10. TI cell programs are enriched in HNSC and associated with disease-free survival

(a) EY-module enrichment in malignant tumors (T) compared to matched normal solid tissues (N) by single sample GSEA among subjects in TCGA-HNSC cohort (n=43 subjects with matched T and N samples). Two-tailed paired T-test: **p<0.01, ****p<0.0001. (b-d) Kaplan-Meier plots for disease-free survival (n=393) among TCGA-HNSC subjects stratified by greater than (red) or less than (blue) median enrichment for the (b) G1/S, (c) pEMT, and (d) Focal Adhesion EY-modules. **Related to Fig. 7.**

REAGENT TABLES

Transgene sequence: HPV16$^{E6\text{-}E7}$

ATGCACCAAAAGAGAACTGCAATGTTTCAGGACCCACAGGAGCGACCCAGAAAGT
TACCACAGTTATGCACAGAGCTGCAAACAACTATACATGATATAATATTAGAATGTG
TGTACTGCAAGCAACAGTTACTGCGACGTGAGGTATATGACTTTGCTTTTCGGGAT
TTATGCATAGTATATAGAGATGGGAATCCATATGCTGTATGTGATAAATGTTTAAAG
TTTTATTCTAAAATTAGTGAGTATAGACATTATTGTTATAGTTTGTATGGAACAACAT
TAGAACAGCAATACAACAAACCGTTGTGTGATTTGTTAATTAGGTGTATTAACTGTC
AAAAGCCACTGTGTCCTGAAGAAAAGCAAAGACATCTGGACAAAAAGCAAAGATTC
CATAATATAAGGGGTCGGTGGACCGGTCGATGTATGTCTTGTTGCAGATCATCAAG
AACACGTAGAGAAACCCAGCTGTAATCATGCATGGAGATACACCTACATTGCATGA
ATATATGTTAGATTTGCAACCAGAGACAACTGATCTCTACTGTTATGAGCAATTAAA
TGACAGCTCAGAGGAGGAGGATGAAATAGATGGTCCAGCTGGACAAGCAGAACCG
GACAGAGCCCATTACAATATTGTAACCTTTTGTTGCAAGTGTGACTCTACGCTTCG
GTTGTGCGTACAAAGCACACACGTAGACATTCGTACTTTGGAAGACCTGTTAATGG
GCACACTAGGAATTGTGTGCCCCATCTGTTCTCAGAAACCATAA

Transgene sequence: YAP1^{S127A} (S127A codon underlined and in bold)

ATGGATCCCGGGCAGCAGCCGCCGCCTCAACCGGCCCCCCAGGGCCAAGGGCA
GCCGCCTTCGCAGCCCCCGCAGGGGCAGGGCCCGCCGTCCGGACCCGGGCAAC
CGGCACCCGCGGCGACCCAGGCGGCGCCGCAGGCACCCCCCGCCGGGCATCAG
ATCGTGCACGTCCGCGGGGACTCGGAGACCGACCTGGAGGCGCTCTTCAACGCC
GTCATGAACCCCAAGACGGCCAACGTGCCCCAGACCGTGCCCATGAGGCTCCGG
AAGCTGCCCGACTCCTTCTTCAAGCCGCCGGAGCCCAAATCCCACTCCCGACAGG
CCAGTACTGATGCAGGCACTGCAGGAGCCCTGACTCCACAGCATGTTCGAGCTCA
T**gCC**TCTCCAGCTTCTCTGCAGTTGGGAGCTGTTTCTCCTGGGACACTGACCCCCA
CTGGAGTAGTCTCTGGCCCAGCAGCTACACCCACAGCTCAGCATCTTCGACAGTC
TTCTTTTGAGATACCTGATGATGTACCTCTGCCAGCAGGTTGGGAGATGGCAAAGA
CATCTTCTGGTCAGAGATACTTCTTAAATCACATCGATCAGACAACAACATGGCAG
GACCCCAGGAAGGCCATGCTGTCCCAGATGAACGTCACAGCCCCCACCAGTCCAC
CAGTGCAGCAGAATATGATGAACTCGGCTTCAGGTCCTCTTCCTGATGGATGGGA
ACAAGCCATGACTCAGGATGGAGAAATTTACTATATAAACCATAAGAACAAGACCA
CCTCTTGGCTAGACCCAAGGCTTGACCCTCGTTTTGCCATGAACCAGAGAATCAGT
CAGAGTGCTCCAGTGAAACAGCCACCACCCCTGGCTCCCCAGAGCCCACAGGGA
GGCGTCATGGGTGGCAGCAACTCCAACCAGCAGCAACAGATGCGACTGCAGCAA
CTGCAGATGGAGAAGGAGAGGCTGCGGCTGAAACAGCAAGAACTGCTTCGGCAG
GCAATGCGGAATATCAATCCCAGCACAGCAAATTCTCCAAAATGTCAGGAGTTAGC
CCTGCGTAGCCAGTTACCAACACTGGAGCAGGATGGTGGGACTCAAAATCCAGTG
TCTTCTCCCGGGATGTCTCAGGAATTGAGAACAATGACGACCAATAGCTCAGATCC
TTTCCTTAACAGTGGCACCTATCACTCTCGAGATGAGAGTACAGACAGTGGACTAA
GCATGAGCAGCTACAGTGTCCCTCGAACCCCAGATGACTTCCTGAACAGTGTGGA
TGAGATGGATACAGGTGATACTATCAACCAAAGCACCCTGCCCTCACAGCAGAAC
CGTTTCCCAGACTACCTTGAAGCCATTCCTGGGACAAATGTGGACCTTGGAACACT
GGAAGGAGATGGAATGAACATAGAAGGAGAGGAGCTGATGCCAAGTCTGCAGGAA
GCTTTGAGTTCTGACATCCTTAATGACATGGAGTCTGTTTTGGCTGCCACCAAGCT
AGATAAAGAAAGCTTTCTTACATGGTTA

Genotyping PCR primers

Oligo name	Sequence	Description	Amplicon Size
K14CreERT_F	CGCATCCCTTTCCAATTTAC		169bp
K14CreERT_R	GGGTCCATGGTGATACAAGG		
Col1_wt_F	TCCCTCACTTCTCATCCAGATATT		
Col1_wt_R	AGTCTTGGATACTCCGTGACCATA	wt:	1092 bp
Col_mut_R	GGACAGGATAAGTATGACATCATCAA	mutant:	480 bp (*YAP1*^{S127A})
Trp53_F	CTTGGAGACATAGCCACACTG	Internal Control	
Trp53_R	TTACACATCCAGCCTCTGTGG		166 bp
Il2_F	CTAGGCCACAGAATTGAAAGATCT	Internal Control	324 bp
Il2_R	GTAGGTGGAAATTCTAGCATCATCC		
HPV16-E67_F2	CTGAGAACAGATGGGGCACA		201 bp
HPV16-E67_R2	GACAGCTCAGAGGAGGAGGA		
rtTA_F	TGCCAACAAGGTTTTTCACTAGAGA		90 bp
rtTA_R	CTCTTGATCTTCCAATACGCAACCTA		
H2B-GFP-F	GCTCGTTTAGTGAACCGTCAG		250 bp
H2B-GFP-R	GACTGTGTCTGATTTCC		
Ctrl_F	AGTGGCCTCTTCCAGAAATGTGC	Internal Control	521 bp
Ctrl_R	TCTTCTGCGCCTTAGTCACC		

LoxP–STOP–LoxP excision assay PCR primers

Oligo name	Sequence	Amplicon Size	Description
LSL_link_pA_F1	GCCTGAAGAACGAGATCAGC	1.5 kb	Intact
LSL_link_recomb_F1	CAAACTCTTCGCGGTCTTTC	400 bp	Recombined
LSL_link_rtTA_R2	AAAATCTTGCCAGCTTTCCCC		

RT-qPCR primers

Oligo name	Sequence	Amplicon Size	Description
hsaYAP1_qPCR_F1	TAGCCCTGCGTAGCCAGTTA	177 bp	Transgene specific Tang et al.
hsaYAP1_qPCR_R1	TCATGCTTAGTCCACTGTCTGT		
HPV16_E6_qPCR_F1	AATGTTTCAGGACCCACAGG	107 bp	Bordigoni et al.
HPV16_E6_qPCR_R1	GTTGCTTGCAGTACACACATTC		
HPV16_E7_qPCR_F1	TCAGAGGAGGAGGATGAAATAGA	111 bp	
HPV16_E7_qPCR_R1	GCACAACCGAAGCGTAGA		
mmuPpia_qPCR_F1	GAGCTGTTTGCAGACAAAGTTC	125 bp	PrimerBank validated oligos
mmuPpia_qPCR_R1	CCCTGGCACATGAATCCTGG		
hEPGN_fwd	ATGGCTTTGGGAGTTCCAATATC	123 bp	PrimerBank validated oligos<br><br>Used to validate EGFR ligand knockdown.
hEPGN_rev	TCCTTCTATGTTGTCAGCTTGC		
hEREG-2_fwd	GGACAGTGCATCTATCTGGTGG	102 bp	
hEREG-2_rev	TTGGTGGACGGTTAAAAAGAAGT		
hNRG1-2_fwd	CGGTGTCCATGCCTTCCAT	160 bp	
hNRG1-2_rev	GGGAGGCTGTTACTGTCATGC		

siRNAs

Name	Description	Supplier	Catalog number	Sequence
siCTRL	Non-targeting Pool	Horizon Discovery	D-001810-10-20	UGGUUUACAUGUCGACUAA
				UGGUUUACAUGUUGUGUGA
				UGGUUUACAUGUUUUCUGA
				UGGUUUACAUGUUUUCCUA
siYAP	Human YAP1 Pool	Horizon Discovery	L-012200-00-0005	GCACCUAUCACUCUCGAGA
				UGAGAACAAUGACGACCAA
				GGUCAGAGAUACUUCUUAA
				CCACCAAGCUAGAUAAAGA
siTAZ	Human WWTR1 Pool	Horizon Discovery	L-016083-00-0005	CCGCAGGGCUCAUGAGUAU
				GGACAAACACCCAUGAACA
				AGGAACAAACGUUGACUUA
				CCAAAUCUCGUGAUGAAUC
siAXL	Human AXL Pool	Horizon Discovery	L-003104-00-0005	ACAGCGAGAUUUAUGACUA
				GGUACCGGCUGGCGUAUCA
				GACGAAAUCCUCUAUGUCA
				GAAGGAGACCCUUAUGGA
siNRG1	Human NRG1 Pool	Horizon Discovery	L-004608-02-0005	UUUCAAACCCCUCGAGAUA
				UUGUAAAAUGUGCGGAGAA
				GGGGAGUGCUUCAUGGUGA
				ACAUCCACCACUGGGACAA
siEREG	Human EREG Pool	Horizon Discovery	L-011268-00-0005	UGACCGUGAUUCUUAUUAU
				GUACAACUGUGAUUCCAUC
				GAAGUGGGUUAUACUGGUG
				GCUCAAGUGUCAAUAACAA
siEPGN	Human EPGN Pool	Horizon Discovery	L-024183-02-0005	AUACAUUGCAAUUGGGAUU
				GGUGUUGGAUUACUAUUAA
				UGUGAGCACUUGACUUUAA
				CGAAGAGGCAGCCGUGACU

IHC antibodies

Primary Antibody	Clone	Dilution	Species	Supplier
Pan-cytokeratin	ab9377	1/200	Rabbit	Abcam
phospho-S6	CST2211	1/400	Rabbit	Cell Signaling Technology
KI67	ab15580	1/400	Rabbit	Abcam
P63	CST39692	1/900	Rabbit	Cell Signaling Technology
SOX2	CST14962	1/300	Rabbit	Cell Signaling Technology

Secondary Antibody	Dilution	Catalog #	Supplier
Goat Anti-Rabbit IgG Antibody (H+L), Biotinylated	1/200	BA-1000	Vector Laboratories

Immunofluorescence antibodies

Primary Antibody	Clone	Dilution	Species	Supplier	Secondary Antibody	Secondary dilution	Secondary catalog #
KRT14	poly19053	1/200	Rabbit	BioLegend	AF568 goat anti-rabbit	1/1000	Thermo A11036
PDPN-biotin	8.1.1	1/100	Syrian Hamster	BioLegend	AF647 streptavidin	1/1000	Thermo A78962
KRT15	Poly18339	1/100	Chicken	BioLegend	AF674 goat anti-chicken	1/1000	Thermo A11036
KI67	ab15580 (poly)	1/200	Rabbit	Abcam	AF568 goat anti-rabbit	1/1000	Thermo A32933

ITGA6	GoH3	1/200	Rat	BioLegend	AF647 goat-anti-rat	1/1000	Thermo A21247
P63	D9L7L	1/200	Rabbit	CST	AF568 goat anti-rabbit	1/1000	Thermo A32933
IBA1	E4O4W	1/200	Rab	CST	AF568 goat anti-rabbit	1/1000	Thermo A32933
LY6G	1A8	1/100	Rat	BioLegend	AF647 goat-anti-rat	1/1000	Thermo A21247
Broad Spectrum Cytokeratin	poly ab86734	1/200	Mouse	Abcam	AF488 goat-anti-mouse	1/1000	Thermo A21121

Flow cytometry antibodies

Antibody	Clone	Dilution	Species	Fluorophore	Supplier, catalog #
CD45	30-F11	1/500	Rat	BUV737	BD, 568344

Immunoblot antibodies

Primary Antibody	Clone	Dilution	Species	Supplier
YAP/TAZ	D24E4	1/1000	Rabbit	Cell Signaling Technology
AXL	C89E7	1/1000	Rabbit	Cell Signaling Technology
CYR61	D4H5D	1/1000	Rabbit	Cell Signaling Technology
pEGFR	1H123	1/1000	Mouse	Cell Signaling Technology
EGFR	D38B1	1/1000	Rabbit	Cell Signaling Technology
pHER3	D1B5	1/1000	Rabbit	Cell Signaling Technology
HER3	D22C5	1/1000	Rabbit	Cell Signaling Technology
pS6	D68F8	1/1000	Rabbit	Cell Signaling Technology
S6	54D2	1/1000	Mouse	Cell Signaling Technology
**Secondary Antibody**		**Dilution**	**Catalog #**	**Supplier**
HRP-goat anti-rabbit IgG		1/10,000	4030-05	Southern Biotechnology
HRP-goat anti-mouse IgG		1/10,000	1030-05	Southern Biotechnology

CUT&Tag antibodies

Primary Antibody	Cat no. / Clone	Dilution	Species	Supplier
Isotype	DA1E	1/50	Rabbit	Cell Signaling Technology
YAP	D8H1X	1/50	Rabbit	Cell Signaling Technology
H3K27ac	D5E4	1/50	Rabbit	Cell Signaling Technology
H3K27me3 (pAb)	Cat no. 39157	1/50	Rabbit	Active Motif
