# Supplementary material for: YAP-driven malignant reprogramming of oral epithelial stem cells at single cell resolution
Source: Nat Commun. 2025 Jan 8;16:498. doi: 10.1038/s41467-024-55660-6 (PMC11711616; doi:10.1038/s41467-024-55660-6)
Supplement: Supplementary file 2 — Description of Additional Supplementary Information [file 41467_2024_55660_MOESM2_ESM.docx]

**SUPPLEMENTARY DATA LEGENDS**

**Supplementary Data 1.** Differentially expressed genes on bulk RNAseq. Tab 1: DESeq2 results for EY vs N p<0.01, log2FC>1. Tab 2: DESeq2 results for Y vs N p<0.01, log2FC>1. Tab 3: DESeq2 results for E vs N p<0.01, log2FC>1. **Related to Fig. 2.**

**Supplementary Data 2.** EY-unique DEGs and gene ontology analysis. Tab 1: List of EY v N unique genes. Tab 2: EY unique MSigDB Hallmarks 2020. Tab 3: EY unique GO processes Panther. **Related to Fig. 2, and Supplementary Fig. 3.**

**Supplementary Data 3.** Gene sets used for GSEA analyses. **Related to Fig 2d-f, 4f, 6a, 7a-f, and Supplementary Fig. 3e, 3g, 6d-f, 7f, 9a.**

**Supplementary Data 4.** EY activated YAP transcriptional targets. Tab 1: List of activated YAP target genes in EY primary cells based on BETA. Tab 2: List of 346 genes with gained YAP binding, local promoter/enhancer activation by H3K27ac, chromatin accessibility, and transcriptional upregulation in EY cells. **Related to Fig. 3d and 3f.**

**Supplementary Data 5.** Epithelial cluster marker genes. Tab 1: cluster 0 - Quiescent Progenitor. Tab 2: cluster 1 - TI cells. Tab 3: cluster 2 - E-enriched. Tab 4: cluster 3 - Differentiating 2. Tab 5: cluster 4 - Differentiating 3. Tab 6: cluster 5 - Cycling Progenitors. Tab 7: cluster 6 - Differentiating 1. Tab 8: cluster 7 - Y-enriched. **Related to Fig. 4.**

**Supplementary Data 6.** Immune cluster marker genes. Tab 1: c0 - CD8 TRM. Tab 2: c1 - Macrophage 1. Tab 3: c2 - PMN-MDSC. Tab 4: c3 - M-MDSC. Tab 5: c4 - CD4 T-cell. Tab 6: c5 - CD4 T-reg. Tab 7: c6 - NK. Tab 8: c7 - Langerhans cells. Tab 9: c8 - Cycling CD8 T-cell. Tab 10: c9 - Macrophage 2. Tab 11: c10 - gd T-cell. Tab 12: c11 - ISG+ CD8+ T cell. Tab 13: c12 - DC. **Related to Fig. 5.**

**Supplementary Data 7.** EY-cluster WGCNA module genes and gene ontology analysis. Tab 1: WGCNA module hub genes and Gene Ontology biological processes by Enrichr. Tab 2: Module 1 genes. Tab 3: Module 2 genes. Tab 4: Module 3 genes. Tab 5: Module 4 genes. Tab 6: Module 5 genes. Tab 7: Module 6 genes. Tab 8: Module 7 genes. Tab 9: Module 8 genes. Tab 10: Module 9 genes. Tab 11: Module 10 genes. Tab 12: Module 11 genes. Tab 13: Module 12 genes. **Related to Fig. 7.**

**Supplementary Data 8.** Uncropped Western blots. **Related to Figure 6 and Supplementary Figure 6.**

**Supplementary Data 9.** Uncropped Agarose gel. **Related to Supplementary Figure 1.**
