# Supplementary figures and images for: YAP-driven malignant reprogramming of oral epithelial stem cells at single cell resolution
Source: Nat Commun. 2025 Jan 8;16:498. doi: 10.1038/s41467-024-55660-6 (PMC11711616; doi:10.1038/s41467-024-55660-6)

**Fig 6d**

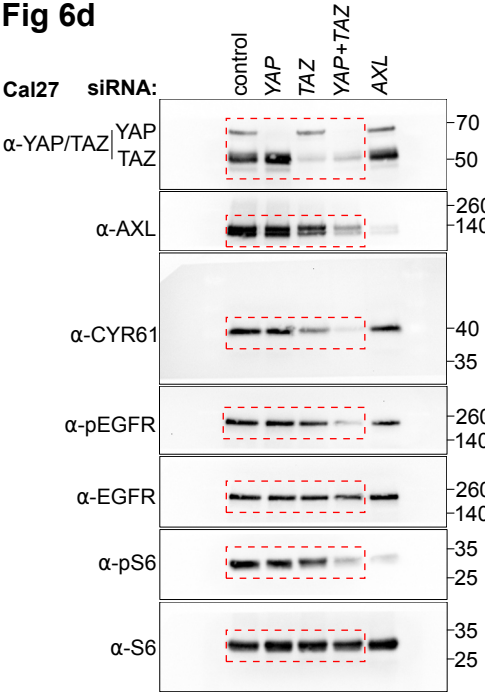

**Fig 6g**

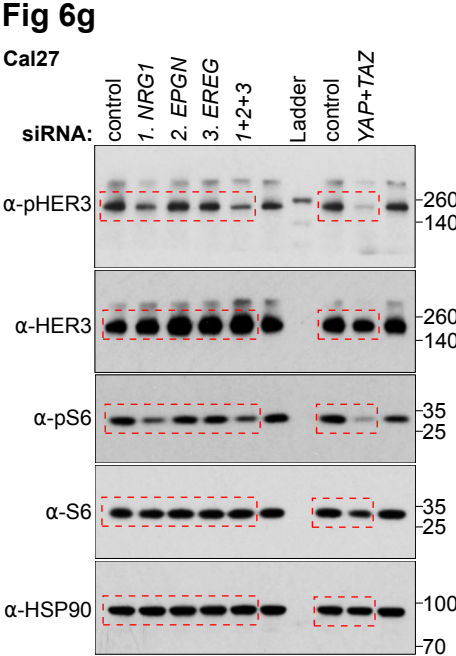

**Fig S9b**

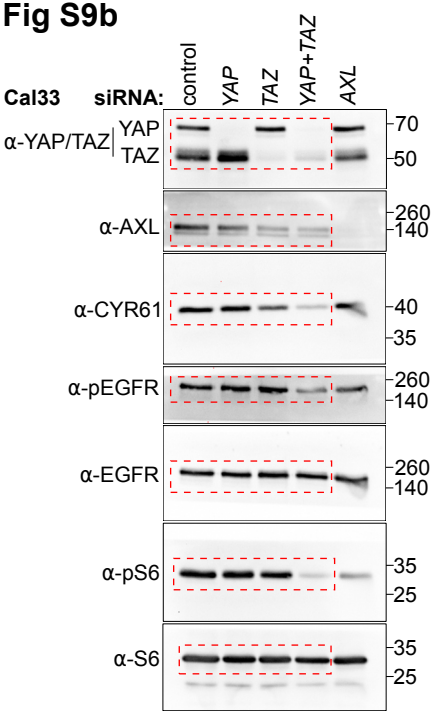

**Fig 6j**

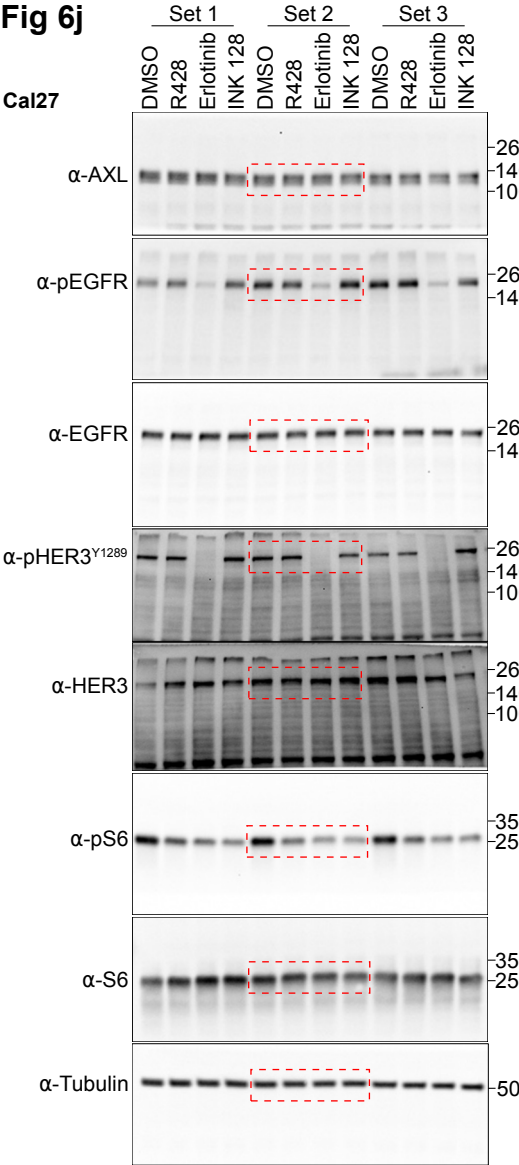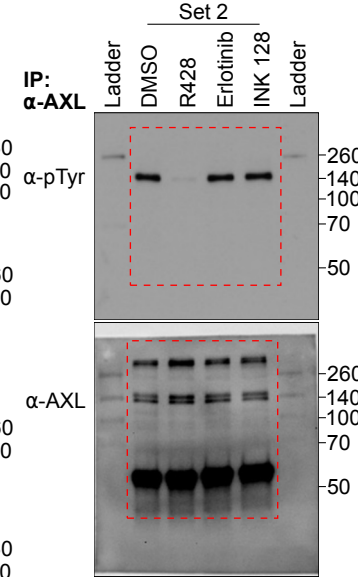

**Fig S9g**

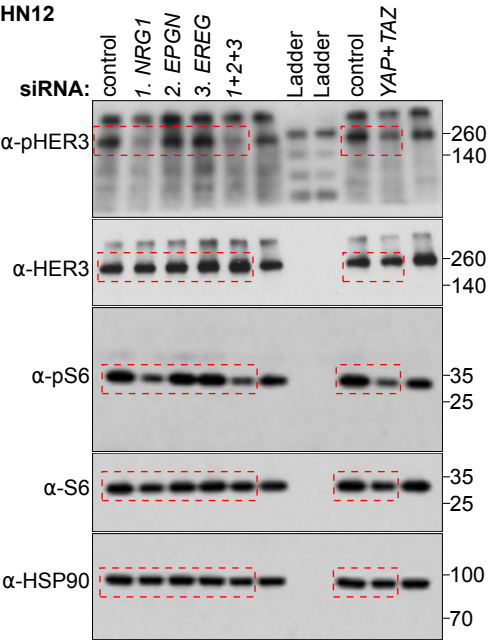

Supplement: Supplementary file 10 — Supplementary Data 8 [file 41467_2024_55660_MOESM10_ESM.pdf]

**Fig S1d**

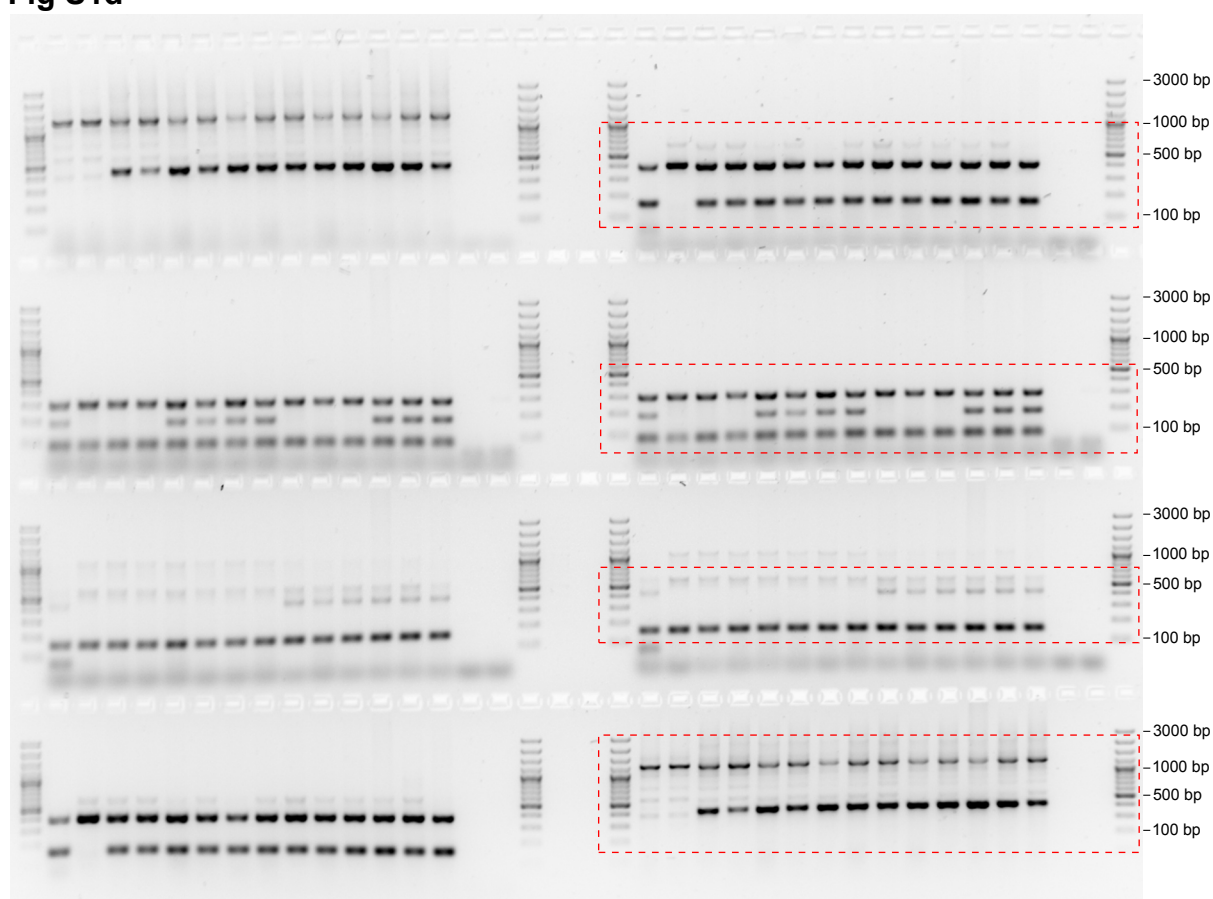

Supplement: Supplementary file 11 — Supplementary Data 9 [file 41467_2024_55660_MOESM11_ESM.pdf]
