# Supplementary material for: YAP-driven malignant reprogramming of oral epithelial stem cells at single cell resolution
Source: Nat Commun. 2025 Jan 8;16:498. doi: 10.1038/s41467-024-55660-6 (PMC11711616; doi:10.1038/s41467-024-55660-6)
Supplement: Supplementary file 12 — Reporting Summary [file 41467_2024_55660_MOESM12_ESM.pdf]

nature portfolio

Corresponding author(s): Farhoud Faraji, J. Silvio Gutkind

Last updated by author(s): Nov 19, 2024

Reporting Summary

Nature Portfolio wishes to improve the reproducibility of the work that we publish. This form provides structure for consistency and transparency in reporting. For further information on Nature Portfolio policies, see our Editorial Policies and the Editorial Policy Checklist.

Statistics

For all statistical analyses, confirm that the following items are present in the figure legend, table legend, main text, or Methods section.

n/a	Confirmed	
☐	☒	The exact sample size (*n*) for each experimental group/condition, given as a discrete number and unit of measurement
☐	☒	A statement on whether measurements were taken from distinct samples or whether the same sample was measured repeatedly
☐	☒	The statistical test(s) used AND whether they are one- or two-sided<br>*Only common tests should be described solely by name; describe more complex techniques in the Methods section.*
☒	☐	A description of all covariates tested
☐	☒	A description of any assumptions or corrections, such as tests of normality and adjustment for multiple comparisons
☐	☒	A full description of the statistical parameters including central tendency (e.g. means) or other basic estimates (e.g. regression coefficient) AND variation (e.g. standard deviation) or associated estimates of uncertainty (e.g. confidence intervals)
☐	☒	For null hypothesis testing, the test statistic (e.g. *F*, *t*, *r*) with confidence intervals, effect sizes, degrees of freedom and *P* value noted<br>*Give P values as exact values whenever suitable.*
☒	☐	For Bayesian analysis, information on the choice of priors and Markov chain Monte Carlo settings
☒	☐	For hierarchical and complex designs, identification of the appropriate level for tests and full reporting of outcomes
☐	☒	Estimates of effect sizes (e.g. Cohen's *d*, Pearson's *r*), indicating how they were calculated

Our web collection on statistics for biologists contains articles on many of the points above.

Software and code

Policy information about availability of computer code

Data collection	For flow cytometry, data was collected using Cytek SpectroFlo (v1 or higher). For FACS, data was collected using BD FACSDiva version 6 or higher.
Data analysis	Bulk RNAseq: Paired-end reads were aligned using STAR v2.7.9 using default settings. STAR index was created using the GRCm39 primary genome FASTA and annotation files. The resulting BAM files were sorted by name using samtools v1.7 then gene counts were quantified using HTSeq-count v0.13.5. Pairwise differential expression was calculated and principal component analysis plots were created using DESeq2 v1.34.0.<br>ATACseq: Raw reads were aligned using BWA (version 0.7.17; PMID: 19451168) to build version mm10 of the mouse genome. Peaks were called using MACS2 (version 2.2.7.1; PMID: 18798982) in narrow peak mode with a False Discovery Rate threshold of less than 0.01. Consensus peaks were merged for all samples by combining all called peaks using bedtools merge (version 2.27.1). Reads were recounted in consensus peaks using bedtools coverage (version 2.27.1). DESeq2 (version 1.42.0) and apeglm (version 1.24.0) in R (version 4.3.2) were used to call differential chromatin accessibility, peaks with adjusted p-value of less than 0.05 were considered significant. Motifs and peak annotation was performed as with the CUT&Tag data using HOMER.<br>CUT&TAGseq: Raw reads were aligned using Bowtie2 (version 2.2.5; PMID: 22388286) to build version mm10 of the mouse genome. Peaks were called independently in each replicate against the corresponding IgG control using Histological images (H&E, IHC, immunofluorescence) were analyzed using QuPath 0.2.3, ImageJ/FIJI, or MATLAB. SEACR (version 1.3, PMID: 31300027) in relaxed mode. Peaks with RPKM < 10 were filtered out. Consensus peaks were merged for each genotype, EY or N, by combining all filtered peaks using bedtools merge (version 2.27.1; PMID: 20110278). Tornado plots were generated using deeptools (version 3.3.5; PMID: 24799436). Differential acetylation was called using DESeq2 (version 1.42.0; PMID: 25516281, PMID: 30395178) and apeglm (version 1.24.0) in R (version 4.3.2). Peaks with adjusted p-values less than 0.05 were considered significant. Motif enrichment was performed using the findMotifsGenome.pl script in the HOMER package (version 4.11; PMID: 20513432). Peaks were annotated using the annotatePeaks.pl script in the HOMER package. Peaks were

annotated if they lie within the gene body or closer than 10 kb to the annotated TSS.

scRNAseq: Single cell gene expression data was processed from the Illumina sequencer files using Cell Ranger (v5.0.0) and its prebuilt mouse reference genome. Individual sample data was then processed and merged using the Seurat (v4.3.0) SCTransform pipeline. Low quality cells (mitochondrial percentage >7, features <1000 and >5500, transcripts per cell >30,000) were filtered prior to data scaling and normalization. After filtering, data was transformed using SCTransform with default parameters, regressing on percent mitochondrial content. Principal component analysis was performed with RunPCA, using the top 50 PCs. Dimensionality reduction was performed with RunUMAP, using the top 30 dimensions. Nearest-neighbor analysis was performed using FindNeighbors using the top 30 dimensions and with k.param set to 50. Clustering was performed with FindClusters with resolution 0.3. Marker genes were calculated using FindAllMarkers with default parameters. Cluster identities were assigned by analysis of differential gene expression.

R package hdWGCNA version 0.2.03 (https://smorabit.github.io/hdWGCNA/) was used for WGCNA analysis in the scRNAseq dataset. Normalization of the integrated Seurat object containing cell-gene expression arrays of EY-genotype epithelial cells was performed using NormalizeMetacells using parameters k=10, max_shared=10, min_cells=20.

Flow cytometry and FACS analyses was performed in FlowJo (BD) version 9 or higher.

For manuscripts utilizing custom algorithms or software that are central to the research but not yet described in published literature, software must be made available to editors and reviewers. We strongly encourage code deposition in a community repository (e.g. GitHub). See the Nature Portfolio guidelines for submitting code & software for further information.

Data

Policy information about availability of data

All manuscripts must include a data availability statement. This statement should provide the following information, where applicable:

- Accession codes, unique identifiers, or web links for publicly available datasets
- A description of any restrictions on data availability
- For clinical datasets or third party data, please ensure that the statement adheres to our policy

The authors confirm that the source data underlying the findings are fully available. Bulk and single cell gene expression, ATAC sequencing, and CUT&Tag sequencing data are available in the NCBI Gene Expression Omnibus database (https://www.ncbi.nlm.nih.gov/geo) under the GEO series records: GSE276778 (ATACseq), GSE276779 (CUT&Tag for YAP, H3K27ac, and H3K27me3), GSE276781 (RNAseq, primary cells); GSE276782 (RNAseq, tissue), GSE276783 (scRNAseq).

Research involving human participants, their data, or biological material

Policy information about studies with human participants or human data. See also policy information about sex, gender (identity/presentation), and sexual orientation and race, ethnicity and racism.

Reporting on sex and gender	All TCGA subjects with available relevant data were included regardless of sex or gender of the participants.
Reporting on race, ethnicity, or other socially relevant groupings	All TCGA subjects with available relevant data were included regardless of race, ethnicity, or other socially relevant groupings of the participants.
Population characteristics	not applicable
Recruitment	not applicable
Ethics oversight	Not applicable. The only human data used in this study was publicly available from The Cancer Genome Atlas (TCGA). https://www.cancer.gov/tcga

Note that full information on the approval of the study protocol must also be provided in the manuscript.

Field-specific reporting

Please select the one below that is the best fit for your research. If you are not sure, read the appropriate sections before making your selection.

☒ Life sciences ☐ Behavioural & social sciences ☐ Ecological, evolutionary & environmental sciences

For a reference copy of the document with all sections, see nature.com/documents/nr-reporting-summary-flat.pdf

Life sciences study design

All studies must disclose on these points even when the disclosure is negative.

Sample size	No statistical method was used to predetermine sample size. Sample sizes for each experiment were determined based on pilot experiments, historical data, and review of the literature, and were determined to be adequate based on the consistency of measurable differences within and between groups.
Data exclusions	No data were excluded from the analyses.
Replication	Every experiment was replicated at least twice with similar results. The manuscript text details that all experimental replicates were biological

	replicates.
Randomization	The following text has been included in the Statistics and Reproducibility statement: “All transgenic mouse experiments were randomized to achieve balanced animal age and sex distributions across experimental conditions.”
Blinding	The investigators were not blinded to allocation during experiments and outcome assessment, except for histopathologic scoring in which pathologists were blinded to the experimental design and conditions.

Reporting for specific materials, systems and methods

We require information from authors about some types of materials, experimental systems and methods used in many studies. Here, indicate whether each material, system or method listed is relevant to your study. If you are not sure if a list item applies to your research, read the appropriate section before selecting a response.

Materials & experimental systems

n/a	Involved in the study
☐	☒ Antibodies
☐	☒ Eukaryotic cell lines
☒	☐ Palaeontology and archaeology
☐	☒ Animals and other organisms
☒	☐ Clinical data
☒	☐ Dual use research of concern
☒	☐ Plants

Methods

n/a	Involved in the study
☒	☐ ChIP-seq
☐	☒ Flow cytometry
☒	☐ MRI-based neuroimaging

Antibodies

Antibodies used

Pan-cytokeratin ab9377 1/200 Rabbit Abcam
phospho-S6 CST2211 1/400 Rabbit Cell Signaling Technology
KI67 ab15580 1/400 Rabbit Abcam
P63 CST39692 1/900 Rabbit Cell Signaling Technology
SOX2 CST14962 1/300 Rabbit Cell Signaling Technology
Goat Anti-Rabbit IgG Antibody (H+L), Biotinylated 1/200 BA-1000 Vector Laboratories

KRT14 poly19053 1/200 Rabbit BioLegend AF568 goat anti-rabbit 1/1000 Thermo A11036
PDPN-biotin 8.1.1 1/100 Syrian Hamster BioLegend AF647 streptavidin 1/1000 Thermo A78962
KRT15 Poly18339 1/100 Chicken BioLegend AF674 goat anti-chicken 1/1000 Thermo A11036
KI67 ab15580 (poly) 1/200 Rabbit Abcam AF568 goat anti-rabbit 1/1000 Thermo A32933
ITGA6 GoH3 1/200 Rat BioLegend AF647 goat-anti-rat 1/1000 Thermo A21247
P63 D9L7L 1/200 Rabbit CST AF568 goat anti-rabbit 1/1000 Thermo A32933
IBA1 E4O4W 1/200 Rab CST AF568 goat anti-rabbit 1/1000 Thermo A32933
LY6G 1A8 1/100 Rat BioLegend AF647 goat-anti-rat 1/1000 Thermo A21247
Broad Spectrum Cytokeratin poly
ab86734 1/200 Mouse Abcam AF488 goat-anti-mouse 1/1000 Thermo
A21121

YAP/TAZ D24E4 1/1000 Rabbit Cell Signaling Technology
AXL C89E7 1/1000 Rabbit Cell Signaling Technology
CYR61 D4H5D 1/1000 Rabbit Cell Signaling Technology
pEGFR 1H123 1/1000 Mouse Cell Signaling Technology
EGFR D38B1 1/1000 Rabbit Cell Signaling Technology
pS6 D68F8 1/1000 Rabbit Cell Signaling Technology
S6 54D2 1/1000 Mouse Cell Signaling Technology

HRP-goat anti-rabbit IgG 1/10,000 4030-05 Southern Biotechnology
HRP-goat anti-mouse IgG 1/10,000 1030-05 Southern Biotechnology

CD45 BUV737 30-F11 1/500 BD 568344

Validation

All antibodies used in this study are commercially available and were validated by the manufacturers. Details regarding the antibodies used and experimental parameters for use are described in the Methods section.

Eukaryotic cell lines

Policy information about cell lines and Sex and Gender in Research

Cell line source(s)	CAL27 (CVCL_1107) and CAL33 (CVCL_1108) cell lines were obtained from the NIDCR Oral and Pharyngeal Cancer Branch cell collection.
Authentication	DNA authentication of cell lines was confirmed by multiplex STR profiling (Genetica DNA Laboratories, Inc. Burlington, NC) to ensure the consistency of cell identity.
Mycoplasma contamination	All cell lines are frequently tested for Mycoplasma contamination. No presence of Mycoplasma was found according to Mycoplasma Detection Kit-Quick Test from Biomake (Houston, TX, USA).
Commonly misidentified lines (See ICLAC register)	No commonly misidentified cell lines were used.

Animals and other research organisms

Policy information about studies involving animals; ARRIVE guidelines recommended for reporting animal research, and Sex and Gender in Research

Laboratory animals	Mice were house in accordance with University of California San Diego Institutional Animal Care and Use Committee (IACUC) guidelines. The UCSD IACUC approved all mouse experiments (Protocol S15195). The following mouse lines were kindly provided by Dr. Elaine Fuchs (The Rockefeller University): Tg(KRT14-cre/ERT)20Efu and Tg(tetO-HIST1H2BJ/GFP)47Efu.19,20 The Col1a1tm1(tetO-Yap1*)Lrsn mouse was kindly provided by Dr. Fernando Camargo (Harvard University). The B6.Cg-Gt(ROSA)26Sortm1(rtTA,EGFP)Nagy/J mouse was obtained from The Jackson Laboratory. The Tg(tetO-HPV16-E6E7)SGu mouse was designed by the Gutkind laboratory and generated in house. NSG™ mice (NOD.Cg-Prkdcscid Il2rgtm1Wjl/SzJ) mice were originally obtained from The Jackson Laboratory and propagated at the Moores Cancer Center. Implantation of transgene epithelial cell suspensions were performed in 8-week-old female NSG mice.
Wild animals	not applicable
Reporting on sex	All transgenic mouse experiments were performed in sex-balanced groups of littermates.
Field-collected samples	not applicable
Ethics oversight	Mice were housed and animal experiments performed in accordance with University of California San Diego Institutional Animal Care and Use Committee (IACUC) guidelines. The UCSD IACUC approved all mouse experiments (Protocol S15195).

Note that full information on the approval of the study protocol must also be provided in the manuscript.

Plants

Seed stocks	not applicable
Novel plant genotypes	not applicable
Authentication	not applicable

Flow Cytometry

Plots

Confirm that:

- [x] The axis labels state the marker and fluorochrome used (e.g. CD4-FITC).
- [x] The axis scales are clearly visible. Include numbers along axes only for bottom left plot of group (a 'group' is an analysis of identical markers).
- [x] All plots are contour plots with outliers or pseudocolor plots.
- [x] A numerical value for number of cells or percentage (with statistics) is provided.

Methodology

Sample preparation	Epithelial cell suspensions were stained for viability using LIVE/DEAD™ Fixable Blue Dead Cell Stain Kit (Thermo #L23105) and BUV737 Rat Anti-Mouse CD45 Clone 30-F11 (BD Biosciences # 568344) and analyzed using a 5-laser Cytek Aurora.<br><br>EY epithelia were isolated and maintained in culture. When the cells were approximately 70-80% confluent, single cell suspensions were generated by subjecting the cells to EDTA and then trypsin, and then mechanically lifting the cells. Cells were counted and viability assessed by trypan blue staining using a Countess III cell counter. The cells were then resuspended in HBSS at a concentration of ~10 million cells/mL and subjected to fluorescence activated cell sorting (FACS) using an Aria II cell sorter. Single cells were identified based on forward and side scatter parameters, and then GFP positive and negative cells were sorted into individual tubes with cell culture medium. Sorted cells were returned to culture and expanded for experimentation and cryopreservation.
Instrument	5-laser Cytek Aurora (Cytek) analyzer, Aria II sorter (BD)
Software	Data were acquired using SpectroFlo (v1 or higher; Cytek) for flow cytometry or FACSDiva (v6 or higher; BD) for FACS. All flow data were analyzed using FlowJo (version 9 or higher).
Cell population abundance	Representative FACS plots with the abundance (percent) of each cell population labeled is shown in Figures S2f and S7d.
Gating strategy	Gating strategies are shown in Figure S2f and Figure S7d.

☒ Tick this box to confirm that a figure exemplifying the gating strategy is provided in the Supplementary Information.
